# A comparison between SOLiD 5500XLand Ion Torrent PGM-derived miRNA expression profiles in two breast cell lines

**DOI:** 10.1590/1678-4685-GMB-2018-0351

**Published:** 2020-04-27

**Authors:** Gabriela Pereira Branco, Renan Valieris, Lucas Venezian Povoa, Luiza Ferreira de Araújo, Gustavo Ribeiro Fernandes, Jorge Estefano Santana de Souza, Maria Galli de Amorim, Elisa Napolitano e Ferreira, Israel Tojal da Silva, Diana Noronha Nunes, Emmanuel Dias-Neto

**Affiliations:** 1 A.C.Camargo Cancer Center, Laboratório de Genômica Médica, CIPE, São Paulo, SP, Brazil.; 2 A.C.Camargo Cancer Center, Laboratório de Biologia Computacional, São Paulo, SP, Brazil.; 3 Instituto Tecnológico de Aeronáutica, Divisão de Ciências Computacionais, Grupo de Inteligência Artificial e Robótica, São José dos Campos, SP, Brazil.; 4 Instituto Federal de Educação, Ciência e Tecnologia de São Paulo, Caraguatatuba, SP, Brazil.; 5 Universidade Federal do Rio Grande do Norte, Instituto Metrópole Digital, Natal, RN, Brazil.; 6 A.C.Camargo Cancer Center, Laboratório de Genômica e Biologia, CIPE, São Paulo, SP, Brazil.; 7 Grupo Fleury Pesquisa e Desenvolvimento, São Paulo, SP, Brazil.; 8 Universidade de São Paulo, Faculdade de Medicina, Departamento & Instituto de Psiquiatria, Laboratório de Neurociências Alzira Denise Hertzog Silva (LIM-27), São Paulo, SP, Brazil.

**Keywords:** Next-generation sequencing (NGS), miRNA expression profiles, SOLiD, PGM

## Abstract

Next-generation sequencing (NGS) platforms allow the analysis of hundreds of millions of molecules in a single sequencing run, revolutionizing many research areas. NGS-based microRNA studies enable expression quantification in unprecedented scale without the limitations of closed-platforms. Yet, whereas a massive amount of data produced by these platforms is available, comparisons of quantification/discovery capabilities between platforms are still lacking. Here we compare two NGS-platforms: SOLiD and PGM, by evaluating their microRNA identification/quantification capabilities using two breast-derived cell-lines. A high expression correlation (R2 > 0.9) was achieved, encompassing 97% of the miRNAs, and the few discrepancies in miRNA counts were attributable to molecules that have very low expression. Quantification divergences indicative of artefactual representation were seen for 14 miRNAs (higher in SOLiD-reads) and another 10 miRNAs more abundant in PGM-data. An inspection of these revealed an increased and statistically significant count of uracyls and uracyl-stretches for PGM-enriched miRNAs, compared to SOLiD and to the miRBase. In parallel, adenines and adenine-stretches were enriched for SOLiDderived miRNA reads. We conclude that, whereas both platforms are overall consistent and can be used interchangeably for microRNA expression studies, particular sequence features appear to be indicative of specific platform bias, and their presence in microRNAs should be considered for database-analyses.

## Introduction

Over the last years, the scientific community produced a remarkable amount of genomic information that helped the understanding of many fundamental medical questions and biological phenomena. This has been made possible by the development of new genomic technologies, which led to dramatic cost-reduction and allowed the deep exploitation of genomes, exomes, and transcriptomes in all areas of biological research (van Dijk *et al.*, 2014).

However, the massive amount of data produced by Next-Generation-Sequencing (NGS) platforms also brought significant challenges regarding solutions for data-storage, analysis, and database management. Furthermore, the use of diverse library construction protocols and sequencing chemistries required by the distinct NGS platforms, resulted in a vast amount of data characterized by high variability in terms of read length, error rates, possible representation biases, and different sequencing error profiles (Shendure and Ji, 2008; Loman *et al.*, 2012; Massingham and Goldman, 2012; Quail *et al.*, 2012; Bragg *et al.*, 2013; Ratan *et al.*, 2013; Yang *et al.*, 2013). This brings an important challenge for the interchangeable use and cross-platform comparison for the assessment-power of publically available data.

Whereas specific databases have been created for the public availability of these reads (e.g., the Sequence Read Archive; www.ncbi.nlm.nih.gov/sra), allowing the public use of the data, studies directed to a systematic comparison of data derived from different NGS platforms are needed to specify platform-dependent discrepancies, sequencing platform biases, and to determine how equivalent and comparable are the data produced by different sequencing strategies. Importantly, the discontinuation of an NGS platform will not preclude or overthrow its data, which will survive and remain useful for later studies.

Although papers have compared miRNA detection and quantification using platforms such as NGS, microarrays, and nCounter (Nanostring) (Willenbrock *et al.*, 2009; Nassirpour *et al.*, 2014; Chatterjee *et al.*, 2015), few manuscripts have systematically compared the sequencing of the same source samples by distinct NGS platforms. Here we compare the composition of microRNA (miRNA) populations derived from two breast cell lines (HB4a and C5.2), as detected by two large-scale sequencing NGS platforms: SOLiD (Sequencing by Oligonucleotide Ligation and Detection) and Ion Torrent PGM (Personal Genome Machine). Both are produced by Thermo Fisher Scientific (USA), and we delve into the analysis of how comparable these datasets are. It is worthy of note that, whereas the Ion Torrent PGM is still in use by many institutions in the world, and the SOLiD platform has been discontinued, thousands of datasets produced by the two are available in public databases, but no cross-comparisons have been published yet.

## Materials and Methods

### Cells

The study was performed with the mammary cell lines HB4a and C5.2. C5.2 is a cell clone derived from the transfection of mammary epithelial origin HB4a with the ERBB2/HER-2 oncogene (Harris *et al.*, 1999). Cells were grown at 37 °C and 5% CO_2_ in RPMI 1640 medium supplemented with 10% fetal bovine serum, 1% antibiotic-antimycotic (penicillin/streptomycin/amphotericin-B; Invitrogen, Carlsbad, CA, USA) and 5 mg/mL hydrocortisone (Sigma-Aldrich, St. Louis, MO, USA) (Carraro *et al.*, 2011).

### RNA extraction and quantification

miRNAs were extracted using the miRNeasy Mini Kit together with the RNeasy MinElute Cleanup Kit in the QIAcube equipment (Qiagen, Hilden, Germany), following the provided instructions. miRNA quantifications were performed using the 2100 Bioanalyzer Small RNA Chip (Agilent, Santa Clara, USA). Aliquots of 100 ng of small RNAs, derived from the same RNA-extraction procedure, were used for the simultaneous preparation of miRNA libraries as follows.

### PGM Ion Torrent miRNA libraries construction and sequencing

For library construction, we followed the protocol of the Ion Total RNA-Seq Kit (Life Technologies, Carlsbad, California, USA). Ion OneTouch 200 Template Kit v2 DL was used for emulsion PCR and sequencing was performed with Ion PGM 200 Sequencing Kit (180 flows).

### SOLID 5500xl miRNA libraries construction and sequencing

The SOLiD Seq Total RNA Kit (Life Technologies, Carlsbad, CA, USA) was used to prepare the miRNA libraries. Some modifications were implemented in the protocol, as follows: (A) Hybridization and RNA binding: miRNAs contained in ≤ 1 *μ*L of enriched small RNAs samples were hybridized and ligated to adapters. For each reaction, we used 3 *μ*L of hybridization solution, plus 2 *μ*L of the SOLiD^TM^ adaptor mix and water to a final volume of 8 *μ*L. The reaction volume was incubated at 65 °C for 10 min and transferred directly to the ice. Subsequently we added 10 *μ*L of 2X ligation buffer and 2 *μ*L of ligation enzyme mix to each reaction, followed by incubation at 16 °C for 16 h. (B) Reverse transcription contained: 4 *μ*L of reverse transcription buffer 10X; 2 *μ*L of dNTP mix (2.5 mM); 2 *μ*L of reverse transcription primer SOLiD^TM^ and 11 *μ*L of nuclease-free water. After incubation at 70 °C for 5 min, we added the 1 *μ*L of the ArrayScript^TM^ Reverse Transcriptase and incubated for 30 min at 42 °C. (C) cDNA purification, size selection and amplification: cDNAs synthesized in the previous step were column-purified with MinElute PCR purification kit (Qiagen, Hilden, Germany). For size selection, 5 *μ*L of the cDNAs were combined with 5 *μ*L of sample buffer (2X Novex® TBE - urea sample buffer), the mixture was heated (95 °C for 3 min) and immediately transferred to ice. Samples were fractionated using the XCell SureLock^TM^ system mini-cell with polyacrylamide gels (10% Novex® TBE Urea Gel 1.0 mM, 10 well) in Novex® TBE running buffer for 1 h at 180 V. The gels were subsequently stained in the same running buffer (1X) containing 5 *μ*L of SYBR® Gold nucleic acid gel stain (Invitrogen) for 10 min. Bands were visualized with the safe blue-light imager transilluminator (Invitrogen) and cDNA fragments ranging from 60 to 70 nt corresponding to miRNAs ligated to adapters were excised and amplified as recommended. Amplicons of two independent PCRs were combined and mixed with 1.8X volumes of the Agencourt^®^ AMPure^®^ XP Beads (Beckman Coulter, Brea, CA, USA) and incubated for 5 min at room temperature. The beads containing amplicons were washed with ethanol, and unbound products were purified again with the same beads (ratio 2:1). Products of interest were eluted in 20 *μ*L of 1X low TE and evaluated with the high-sensitivity bioanalyzer chip, as recommended in the protocol. (D) The E20 emulsion PCR (ePCR) and ePCR enrichment were performed following the recommended protocols (Applied Biosystems, Foster City, CA, USA). Sequencing was performed according to the protocol 5500 Series Genetic Analysis System User Guide (Applied Biosystems).

### Bioinformatics and statistical analyses

Sequencing quality was good for both platforms, exceeding Q20-30 for a length above the average miRNA size. miRNAs were identified from both SOLiDand PGM-derived reads after quality filtering and adapter removal, using the default parameters of miRDeep2 version

2.0.05 (Friedländer *et al.*, 2012). Next, filtered reads were mapped against miRBase (release 22) using the same miRDeep2 version. Default parameters were used allowing a maximum of one mismatch and, a seed sequence of 18 nt without mismatches. Due to intrinsic differences of PGM and SOLiD, which have very distinct throughputs (SOLiD yielded ~4 times more sequences than PGM), we applied a stringent requirement for considering the presence of specific miRNAs in this dataset. Therefore, in order to compare the miRNAs represented by each NGS platform, a miRNA was considered if at least two miRNA-corresponding reads were available from PGM-data and at least eight reads were derived from SOLiD (4x difference throughput correction coefficient, applied to consider the coverage achieved here for the different platforms).

To avoid mapping the sequencing reads to miRNAs that are indeed distinct molecules and that differ from each other by a single nucleotide, mature miRNAs were pairwise aligned using a local sequence alignment tool (EMBOSS; Water 6.5.7) (Rice *et al.*, 2000). miRNAs with no or with a single mismatch were clustered together (Table S1). Read counts were calculated for each miRNA (clusters or unique) and after sequence alignment and annotation, R (v2.12) and PERL (v5.14.2) scripts were applied to standardize miRNA nomenclature and counts across all samples. We evaluated the expression correlation and differential representation of the miRNAs identified by SOLiD and PGM using Variance Stabilizing Transformation (VST) of expression as a means to have precise correlation matrices, and to verify if expression levels could affect the differential representation between these platforms.

Saturation/rarefaction curves were built using all mature miRNAs identified by a sampling subset of reads from 1% to 100% for each sample. Correlation curves were drawn using log2 transformation of the number of reads normalized per million-reads sequenced in each sample. Outliers were considered when the normalized count difference between samples was greater than the 1.5*IQR + Q3 value, where IQR is the interquartile range (Q3-Q1) and Q1 and Q3 are the first and third quartiles. Nucleotide content and homopolymer counts were performed in Excel Statistical analysis (*t*-test) and boxplots were generated in R environment (v. 3.5.1) using the packages R Commander and ggplot2, respectively (Fox and Bouchet-Valat, 2018).

In order to verify structural composition differences for miRNAs identified in one or another NGS platform, we represented each miRNA as directed and undirected graphs. A nucleotide was described as a node, while an edge was created between adjacent nucleotides. Each edge received a weight corresponding to the number of times that two nucleotides appear next to each other. Based on that representation, we computed the degree (i.e., number of edges connected to a node) of each nucleotide. Then, we applied the Z-test, adjusted by the Benjamini and Hochberg method to compare the average degree between miRNAs of each platform, contrasting with the entire miRBase. As we represented a miRNA as both directed and undirected graph, our approach generated three degree values for each nucleotide. With this, we could classify edges as incoming (in), outgoing (out), and undirected (both), resulting in 12 degree groups (e.g., a_in, c_out, g_both, u_in, etc). For each miRNA we built a directed graph representation. Each node corresponds to one of the four possible nucleotides, whereas the adjacent nucleotides are represented by the edges (upstream and downstream). Overplotting of all miRNAs of a certain source (PGM-enriched, SOLiD-enriched or the whole miRBase) was used to generate a representation of these sets and to investigate degree differences. Over-represented edges were considered when statistical differences (*p* < 0.05) were observed. Plots and analyses were performed using NetworkX (Hagberg *et al.*, 2008), Python 3, and R (v3.5.1). *P*-values < 0.05 were considered to be statistically significant.

## Results

After quality and size filtering we evaluated 7,883,393 reads provided by SOLiD and 1,924,046 reads provided by the Ion PGM (4.1X difference). Both platforms gave good quality reads (average above Q30) in the size range of miRNAs. The percentage of miRbase mapped and unmapped reads, and sequencing reads removed due to low quality, for both cell lines and platforms is given in Figure S1. Table 1 shows the number of reads and the set of expressed miRNAs determined by miRDeep2 for both cell lines by these two sequencing platforms, including the number of distinct miRNAs identified by both platforms for both cell lines, as well platform-specific miRNAs.

**Table 1:** Sequencing results and miRNA identification in cell lines for NGS platforms.

Platforms	HB4a		C5.2
Valid reads (PGM)	1,099,181		824,865
Valid reads (SOLiD)	3,501,788		4,381,605
Distinct miRNAs identified by PGM^a^	416		429
Distinct miRNAs identified by SOLiD^b^	407		438
Total distinct miRNAs (SOLiD + PGM)	465		495
miRNAs more abundant in PGM^c^	3 (0.6%)	3 (0.6%)	4 (0.8%)
miRNAs more abundant in SOLiD^d^	3 (0.6%)	6 (1.3%)	5 (1.1%)

^a^Considering only miRNAs identified by at least 2 reads. ^b^Considering only miRNAs identified by at least 8 reads. ^c^Considering a total of 491 distinct miRNAs identified in both cell lines using PGM, there are 10 miRNAs over-represented by PGM, being 3 present in both HB4a and C5.2. ^d^Considering a total of 478 distinct miRNAs identified in both cell lines by SOLiD, there are 14 miRNAs over-represented, being 6 found in both cell lines.

**Figure 1:**
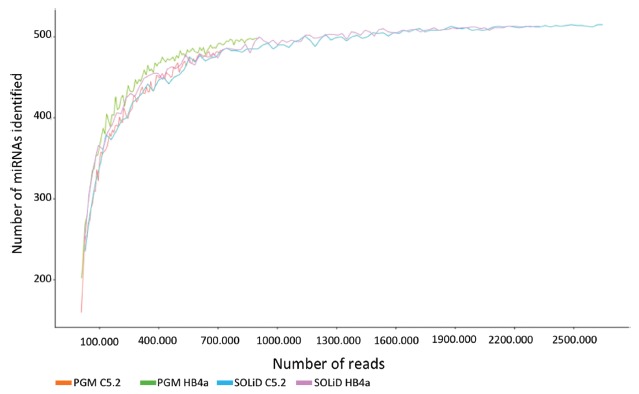
Saturation plots of miRNAs derived from HB4a and C5.2 cell lines, as identified by SOLiD and PGM.

Besides the inherent throughput differences from the Ion-PGM and the SOLiD NGS platforms, we observed very similar trends for the saturation profiles (Figure 1), which suggest a similar number of miRNAs (~500) expressed by both cell lines.

**Figure 2:**
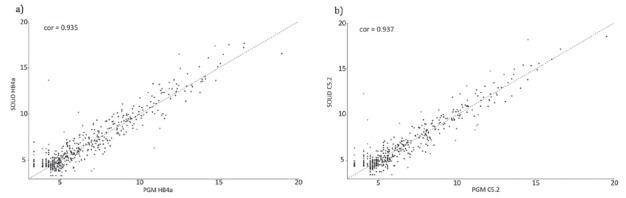
Plots showing the miRNA expression-correlation analysis between SOLiD and PGM platforms for the cell lines HB4a (COR=0.935) (a) and C5.2 (COR=0.937) (b). Expression levels for each miRNA are given in normalized counts calculated by DESeq2 variance-stabilizing transformation. Differentially represented miRNAs calculated by DESeq2 are indicated in red. The Pearson correlation coefficient (cor) was calculated for each cell line.

**Figure 3:**
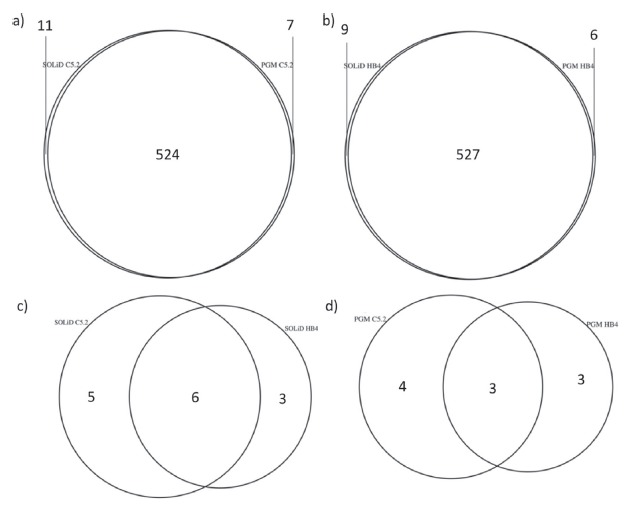
Venn diagrams depicting the sets of miRNAs preferentially represented in C5.2 (a), or HB4a (b) cell lines using SOLiD or PGM platforms. miRNAs more abundantly represented in SOLiD (c), or PGM (d). The miRNAs indicated here correspond to those indicated by red dots in Figure 2 and to the last two lines of Table 1.

Our analyses demonstrate that both sequencing platforms allow a robust representation of miRNAs in terms of saturation, number, and abundance of the identified miRNAs. Good expression correlations, as determined by VST, were found between these platforms for both cells (Figure 2 **;** R^2^ > 92). High similarities between these platforms could also be seen in the Venn diagrams (Figure 3) that show the presence of the majority of miRNAs to be indicated by both platforms, for both cell lines.

The clustering of mature miRNAs with no or a single mismatch resulted in 39 distinct clusters, encompassing 102 miRNAs (Table S1). Our analyses of representation correlates also show that most miRNAs that are differentially represented between platforms have low levels of expression (70% and 72% are below the size factor normalization) (Table S2), respectively for HB4a and C5.2 (Figure 2) (Anders and Huber, 2010). However, a few very discrepant cases can also be seen, including the most highly divergent miRNA identified here, hsa-miR-3607-5p, with 3 and 1 counts per million in PGM versus 5354 and 2094 counts in SOLiD, for HB4a and for C5.2 respectively (Tables 2 and 3).

**Table 2:** miRNAs more abundantly detected by the SOLiD platform.

miRNA (hsa-miR)	Fold change	p-value	Reads/million	Reads/million	Cell line	Sequence
	(SOLiD:PGM)		(SOLiD)	(PGM)		
150-5p^a^	N.A.	0.0084	26.02	0	C5.2	ucucccaacccuuguaccagug
	26.36x	0.0028	23.99	0.91	HB4a	
142-5p^b^	N.A.	0.0302	12.57	0	HB4a	cauaaaguagaaagcacuacu
	10.7x	0.1141	12.01	1.12	C5.2	
223-3p^b^	N.A.	0.0340	12.55	0	C5.2	Ugucaguuugucaaauacccca
	N.A.	0.1535	5.14	0	HB4a	
3607-5p^1^	1731x	0.0367	2094.4	1.21	C5.2	gcaugugaugaagcaaaucagu
	1968x	0.0286	5353.8	2.73	HB4a	
4284^a^	118.8x	0.0002	287.57	2.42	C5.2	gggcucacaucaccccau
	14.39x	0.0042	471.19	32.75	HB4a	
199a-3p/ 199b-3p^a^	17.6x	0.0429	21.23	1.21	C5.2	acaguagucugcacauugguua
	12.86x	0.0274	35.12	2.73	HB4a	
1249^b^	16.13x	0.0180	39.03	2.42	C5.2	acgcccuucccccccuucuuca
	N.A.	0.2007	43.98	0	HB4a	
181b-3p^b^	13.18x	0.0424	23.99	1.82	HB4a	cucacugaacaaugaaugcaa
	1.69x	0.7040	2.05	1.21	C5.2	
29a-3p/ 29c-3p^a^	4.19x	0.0266	11927	2849	C5.2	uagcaccaucugaaaucgguua
	10.48x	0.0062	37988	3622	HB4a	uagcaccauuugaaaucgguua
103a-3p^a^	8.41x	0.0060	128212	15233	C5.2	agcagcauuguacagggcuauga
	4.03x	0.0406	70822	17552	HB4a	
152-5p^b^	8.07x	0.0202	102.8	12.74	HB4a	agguucugugauacacuccgacu
	7.78x	0.0517	28.30	3.64	C5.2	
4521^b^	5.61x	0.0200	217.73	38.79	C5.2	gcuaaggaaguccugugcucag
	2.99	0.0842	261.30	87.34	HB4a	
301b^b^	4.39x	0.0270	532.68	121.23	C5.2	cagugcaaugauauugucaaagc
	2.06x	0.1569	589.98	286.58	HB4a	
107^b^	3.82x	0.0324	3831.24	1000.16	C5.2	agcagcauuguacagggcuauca
	2.55x	0.0999	3918.28	1535.69	HB4a	

^a^ miRNAs preferentially represented by the SOLiD platform for both cell lines; ^b^ significant differential representation seen for only one of the cell lines. For each miRNA row, the top line contains the lower *p*-value for differential representation between SOLiD and PGM.

**Table 3:** miRNAs more abundantly detected by the PGM platform.

miRNA (hsa-miR)	Fold change	p-value	Reads/million	Reads/million	Cell line	Sequence
	(PGM: SOLiD)		(PGM)	(SOLiD)		
3613-5p^b^	N.A.	0.0062	24.25	0	C5.2	uguuguacuuuuuuuuuuguuc
	25.52x	0.1131	14.55	0.57	HB4a	
4455^b^	N.A.	0.0472	10.92	0	HB4a	aggguguguguguuuuu
	N.A.	N.A.	0	0	C5.2	
424-3p^b^	118.1	0.0270	67.32	0.57	HB4a	cagugcaaugauauugucaaagc
	6.76x	0.1348	50.92	7.53	C5.2	
16-1-3p^a^	67.03x	0.0028	76.42	1.14	HB4a	ucucccaacccuuguaccagug
	45.13x	0.0084	61.83	1.37	C5.2	
25-5p^b^	46.28x	0.0340	26.38	0.57	HB4a	ugucaguuugucaaauacccca
	19.99x	0.0873	18.19	0.91	C5.2	
20a-3p^a^	48.37x	0.0274	1215.45	25.13	HB4a	acaguagucugcacauugguua
	11.08x	0.0429	1446.3	130.55	C5.2	
let-7i-5p^a^	8.16x	0.0060	1659.7	203.35	C5.2	agcagcauuguacagggcuauga
	11.43x	0.0406	1563.0	136.79	HB4a	
1296-5p^b^	12.75x	0.0324	189.12	14.83	C5.2	uuagggcccuggcuccaucucc
	8.62x	0.0797	118.27	13.71	HB4a	
200c-3p^b^	11.23x	0.0202	602.52	53.63	C5.2	agguucugugauacacuccgacu
	5.27x	0.1320	2037.88	386.66	HB4a	
1307-5p^b^	8.59x	0.0180	1574.8	183.27	C5.2	acgcccuucccccccuucuuca
	6.55x	0.0872	1924.16	293.56	HB4a	

^(a)^ miRNAs preferentially represented by the PGM platform for both cell lines; ^(b)^ miRNAs with significant differential representation observed for a single cell line. For each miRNA row, the top line contains the lower p-value for differential representation between SOLiD and PGM.

Our results are in agreement with the expected similarity of HB4a and its derived clone C5.2, which differ only in respect to the overexpression of *ERBB2* in the latter, and show a very similar miRNA expression profile for both cells. Using the criteria adopted here, we found exactly the same number of expressed miRNAs (542 Figure 3a,b) for both cell lines. Out of these, a small fraction of 2.76% and 3.3% were differentially represented by one or another NGS platforms (respectively in HB4a and C5.2). The full list of miRNAs identified for both cell lines, as well as their normalized level of expression (given in RPKM - Reads Per Kilo base Per Million - which, for normalization, considers transcript length and sequencing depth) for both platforms is given in Table S3. Lists of miRNAs with significant differences in representation by one or another NGS platform are given in Tables 2 and 3.

Our next step was to investigate which factors could have impacted the differential representation of miRNAs by these platforms. As the chemistry used by the PGM is prone to errors in homopolymeric regions, we investigated the effect of repeats of 2, 3, or 4 nt on the differentially represented miRNAs. However, no significant differences were found. We further evaluated whether the primary sequences of these miRNAs would contain discrepant amounts of any of the bases or GC content. We found that the miRNAs over-represented by PGM had higher content of uracyls (34.4% in PGM x 25% in SOLiD; *p*=0.047) whereas miRNAs with increased levels in SOLiD were richer in adenines (28.9% in SOLiD x 19.2% in PGM; *p*=0.0363) (Figure S2, Table S4).

**Figure 4:**
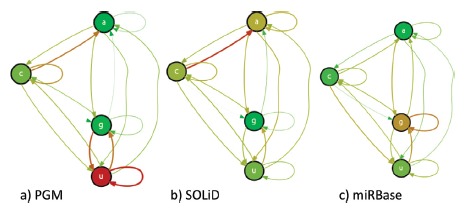
Overplot of all graphs from the differentially represented miRNAs found in PGM (a) and SOLiD (b), compared to the overall compositional trend of all miRNAs available in miRBase (c). Both size and color of a node or edge describe their weights, where thin green and thick red representations describe the minimum and maximum number of connections each graph, respectively.

**Figure 5:**
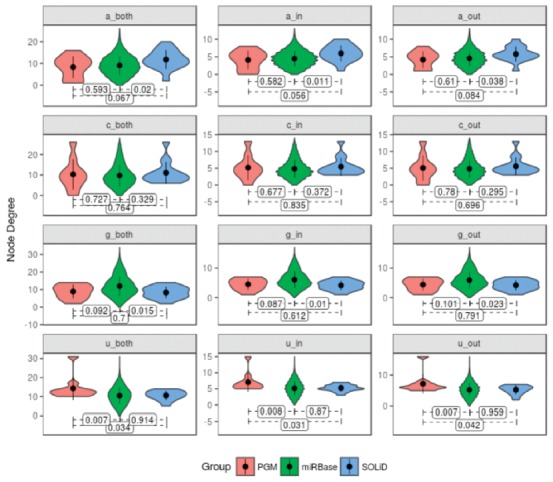
*P*-values of Z-tests comparing average node degree of PGM and miRBase and SOLiD and miRNA sequences. Labels on X-axis describe node label (nucleotide) at prefix and edge type at suffix.

As we continued to evaluate if the structural composition of the mature miRNA sequences could interfere with its representation in the NGS platforms, we considered compositional biases involving continuous stretches of nucleotides, where a nucleotide is followed, preceded or found in continuous homogeneous stretches. The overplotting of the compositional bias of PGM-, SOLiD-, or the overall miRBase composition revealed some significant trends (Figure 4). A careful analysis showed a significant bias for continuous uracyl stretches in PGM compared to SOLiD, as well as to miRBase (respectively *p*-values of 0.007 and 0.031), a finding that could indicate an overrepresentation of these miRNAs in PGM-derived data. For the set of differentially represented miRNAs in PGM, node degrees were statistically different for all uracil nucleobase edge types (Figure 5).

Different trends, with higher but still significant *p*-values, were found for miRNAs differentially represented by the SOLiD platform. A comparison of these against the composition of miRBase and PGM-over represented miRNAs resulted in respective *p*-values of 0.011 to 0.056 for all adenine edge types, respectively. Further, comparisons of PGM with miRBase resulted in a *p*-value of 0.582. Accordingly, node degrees are statistically different for all uracil nucleobase edge types evaluating SOLiD opposite to PGM.

We applied hypothesis tests assuming alternative *H*_*A*_ *= μ* ≠ *μ0* with a 5% significance level for the differential trends of nucleotide connections depicted in Figure 4. The results, given in Figure 5 compact average, standard deviation, and probability density of node degrees for each edge type and miRNA group by means of a violin plot. *P*-values of pairwise Z-tests are shown at the bottom of each subplot. Subplot headers identify their corresponding edge type.

## Discussion

In the field of transcriptome research, when the aim is to determine differentially expressed transcripts, the many available NGS platforms provide a very appealing approach due to their intrinsic openness (where the transcripts to be evaluated are not restricted to those available in closed platforms, such as qRT-PCR or hybridization arrays), dynamic range of detection, and other benefits.

NGS technologies have strongly impacted genomics and will have far-reaching value for many areas of biological and biomedical research. In this sense, the high coverage and accuracy provided by NGS is likely to make the data useful for many years to come. Therefore, it is relevant to mention that a brief analysis of the NCBI Short Reads Archive (SRA November, 2018) revealed 1,314 miRNA studies using SOLiD and another 1,349 (1096 + 253 respectively for the terms Ion Torrent or PGM and miRNA) for PGM-derived data. Each one of these contains several unique experiments, including different approaches and covering diverse species.

There are intrinsic advantages given by NGS to study miRNAs, such as the identification of mutations, polymorphisms, miRNA-editing, expression levels, and even the identification of new miRNAs (Friedländer *et al.*, 2008; Creighton *et al.*, 2009; Huang *et al.*, 2009; Lu *et al.*, 2009; Wei *et al.*, 2009; Meiri *et al.*, 2010; Sung *et al.*, 2016). Hence, this approach is rapidly replacing others, such as RT-qPCR arrays and microarrays. However, the full transcriptional characterization of miRNAs has been partially limited by the complexity and increased time requirements of available RNA-seq library construction methods used in NGS, which also seem to have a systematically biased representation of miRNAs (Linsen *et al.*, 2009; Tian *et al.*, 2010; Hafner *et al.*, 2011; Van Nieuwerburgh *et al.*, 2011; Leshkowitz *et al.*, 2013)**.** These trends may be introduced during PCR amplification, ligation, and cDNA synthesis steps (Leshkowitz *et al.*, 2013).

The chemistries used by SOLiD and Ion PGM platforms have been described elsewhere (Heather and Chain, 2016). In brief, the SOLiD makes use of sequencing-byligation steps in which an emulsion-PCR (ePCR) with small magnetic beads is used to amplify the DNA fragments for parallel sequencing. Each ligation step is followed by fluorescence detection and another round of ligation, allowing between 80-100 Gbp of sequences to be produced per run, or over 2 billion reads per run with a raw base accuracy of 99.94% due to its 2-base encoding mechanism. Besides its high throughput and accuracy, this technology is no longer in use, as it is extremely laborious, and almost a month is required to perform a sequencing run.

The Ion Personal Genome Machine (PGM) was the first available NGS platform that uses no fluorescence or image capture (Liu *et al.*, 2012). The sequencing is based on the use of a semiconductor chip that detects the reduction of pH when an ion proton is released right after the incorporation of a nucleotide by the polymerase. The system is capable of producing longer reads (up to 400nt) and it is fast (2-4 hour runs) (Liu *et al.*, 2012; Quail *et al.*, 2012). The use of this platform has grown recently, especially for clinical applications, small laboratories, and for the investigation of less-diverse transcriptomes and smaller genomes (Liu *et al.*, 2012). However, PGM presents significant homopolymer-associated indel errors (1.5 errors per 100 bases) (Loman *et al.*, 2012), which may affect the correct identification or the mapping of shorter molecules such as miRNAs.

Our comparison of the PGM and SOLiD platforms showed a high quantitative correlation between these platforms for two independent cell lines with similar trends in saturation levels, and > 97% of the miRNAs were detected with no significant quantitative differences. This indicates the capability of SOLiD and PGM to provide a robust representation of miRNAs with very few miRNAs showing quantitative discrepancies. After a series of analyses, our data suggest that the representation of miRNAs with continuous uracyl stretches is likely to be inflated by PGM, as the levels of these are higher when compared to miRNAs found by SOLID for the same cell lines and is also above the levels found for the whole miRBase, suggesting an artificial enrichment of these sequences by PGM. On the other hand, we also found evidence that average degrees of nodes representing continuous stretches of adenines are enriched for the SOLiD data as compared to miRBase, but non significant *p*-values found when compared to PGM. In this sense, we recommend caution when using miRNA databases, especially when PGM-derived data would suggest uracyl-enriched miRNAs to be over-represented in a particular dataset, compared to a non-PGM set of data.

The sharing of scientific data in general, and the deposition of DNA/RNA sequencing data in particular, is a practice that needs to be constantly fostered and reinforced by journals, academia, and funding agencies that support research. The comparison and/or validation of data using public databases of nucleotide sequences, including the investigation of miRNA expression patterns, is an important source of additional information that may reveal important scientific findings.

## Supplementary material

The following online material is available for this article:

Figure S1 - Distribution of filtered reads from PGM and SOLiD platforms for both cell lines.

Figure S2 - Box plots comparing nucleotide content and nucleotide repeats longer than 2, 3 and 4 between the most abundant miRNAs found by PGM and Solid platform.

Table S1 - miRNAs clusters.

Table S2 - Size factor used for normalization.

Table S3 - List of miRNAs identified for both cell lines in PGM and SOLiD.

Table S4 - Homopolymers in miRNAs identified by PGM and SOLiD.
