# Supplementary material for: A comparison between SOLiD 5500XLand Ion Torrent PGM-derived miRNA expression profiles in two breast cell lines
Source: Genet Mol Biol. 2020 Apr 27;43(2):e20180351. doi: 10.1590/1678-4685-GMB-2018-0351 (PMC7201575; doi:10.1590/1678-4685-GMB-2018-0351)
Supplement: Figure S1 - [file 1415-4757-GMB-43-2-e20180351-suppl1.pdf]

Supplementary Material to "A comparison between SOLiD 5500XL- and Ion Torrent PGM-derived miRNA expression profiles in two breast cell lines"

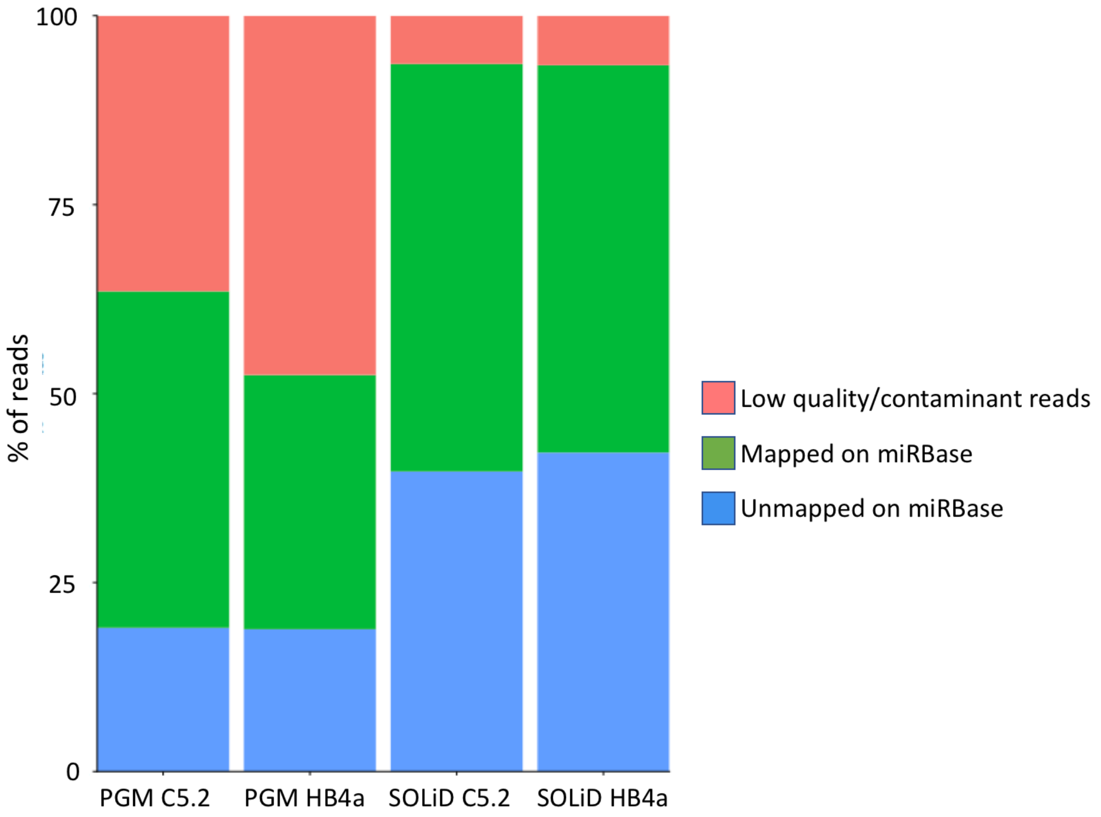

Figure S1 - Distribution of filtered reads from PGM and SOLiD platforms for both cell lines.
