# Supplementary material for: A comparison between SOLiD 5500XLand Ion Torrent PGM-derived miRNA expression profiles in two breast cell lines
Source: Genet Mol Biol. 2020 Apr 27;43(2):e20180351. doi: 10.1590/1678-4685-GMB-2018-0351 (PMC7201575; doi:10.1590/1678-4685-GMB-2018-0351)
Supplement: Figure S2 - [file 1415-4757-GMB-43-2-e20180351-suppl2.pdf]

Supplementary Material to "A comparison between SOLiD 5500XL- and Ion Torrent PGM-derived miRNA expression profiles in two breast cell lines"

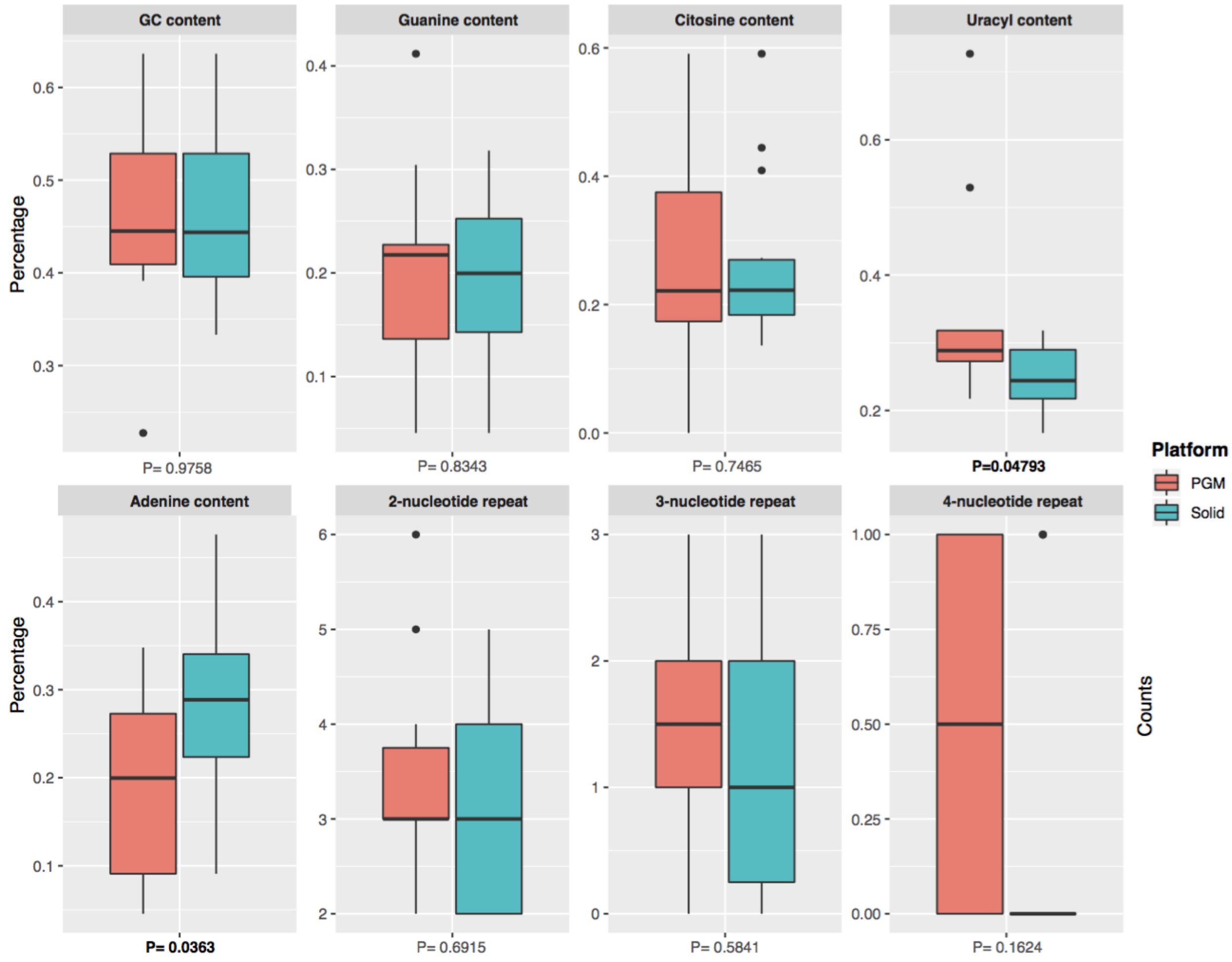

Figure S2 - Box plots comparing nucleotide content and nucleotide repeats longer than 2, 3 and 4 between the most abundant miRNAs found by PGM and Solid platform. Y-axis in the first five plots refer to nucleotide percentage and Y-axis in the last three plots refer to counts. P-values for the comparisons can be found below each plot.
