# Supplementary material for: A comparison between SOLiD 5500XLand Ion Torrent PGM-derived miRNA expression profiles in two breast cell lines
Source: Genet Mol Biol. 2020 Apr 27;43(2):e20180351. doi: 10.1590/1678-4685-GMB-2018-0351 (PMC7201575; doi:10.1590/1678-4685-GMB-2018-0351)
Supplement: Table S1 - [file 1415-4757-GMB-43-2-e20180351-suppl3.pdf]

Supplementary Material to "A comparison between SOLiD 5500XL- and Ion Torrent PGM-derived miRNA expression profiles in two breast cell lines"

Table S1 – miRNAs clusters.

Clusters	miRNAs
1	hsa-miR-548ai; hsa-miR-570-5p
2	hsa-miR-548ah-3p; hsa-miR-548aq-3p; hsa-miR-548am-3p
3	hsa-miR-3689b-5p; hsa-miR-3689b-5p; hsa-miR-3689a-5p
4	hsa-miR-133a-3p; hsa-miR-133b
5	hsa-miR-517c-3p; hsa-miR-517b-3p; hsa-miR-517a-3p
6	hsa-miR-30e-3p; hsa-miR-30a-3p
7	hsa-miR-17-5p; hsa-miR-106a-5p
8	hsa-miR-99a-5p; hsa-miR-100-5p
9	hsa-miR-30e-5p; hsa-miR-30a-5p; hsa-miR-30d-5p
10	hsa-miR-520c-3p; hsa-miR-520f-3p
11	hsa-miR-29c-3p; hsa-miR-29a-3p
12	hsa-miR-520d-5p; hsa-miR-527; hsa-miR-518a-5p
13	hsa-miR-516a-3p; hsa-miR-516b-3p
14	hsa-miR-520c-5p; hsa-miR-519c-5p; hsa-miR-526[a]; hsa-miR-522-5p; hsa-miR-519a-5p; hsa-miR-518d-5p; hsa-miR-518f-5p; hsa-miR-523-5p; hsa-miR-519b-5p; hsa-miR-518e-5p
15	hsa-miR-3689c; hsa-miR-3689a-3p; hsa-miR-3689b-3p
16	hsa-miR-23a-3p; hsa-miR-23b-3p
17	hsa-miR-513c-3p; hsa-miR-513a-3p
18	hsa-miR-18b-5p; hsa-miR-18a-5p
19	hsa-miR-548t-3p; hsa-miR-548aa
20	hsa-miR-548i; hsa-miR-548c-5p; hsa-miR-548b-5p; hsa-miR-548d-5p; hsa-miR-548o-5p; hsa-miR-548as-5p; hsa-miR-548am-5p
21	hsa-miR-196b-5p; hsa-miR-196a-5p
22	hsa-miR-4520b-5p; hsa-miR-4520a-5p

**Clusters**	**miRNAs**
23	hsa-let-7c-5p; hsa-let-7a-5p; hsa-let-7e-5p; hsa-let-7f-5p; hsa-let-7b-5p
24	hsa-miR-19b-3p; hsa-miR-19a-3p
25	hsa-miR-4662b; hsa-miR-4662a-3p
26	hsa-miR-520e; hsa-miR-520b
27	hsa-miR-129-2-3p; hsa-miR-129-1-3p
28	hsa-miR-548au-5p; hsa-miR-548ay-5p; hsa-miR-548ar-5p
29	hsa-miR-10b-5p; hsa-miR-10a-5p
30	hsa-miR-181b-5p; hsa-miR-181d-5p
31	hsa-miR-199b-3p; hsa-miR-199a-3p
32	hsa-miR-27b-3p; hsa-miR-27a-3p
33	hsa-miR-642b-3p; hsa-miR-642a-3p
34	hsa-miR-135a-5p; hsa-miR-135b-5p
35	hsa-miR-1185-2-3p; hsa-miR-1185-1-3p
37	hsa-miR-548aj-5p; hsa-miR-548x-5p; hsa-miR-548g-5p
37	hsa-miR-548z; hsa-miR-548h-3p
38	hsa-miR-365b-3p; hsa-miR-365a-3p
39	hsa-miR-548ai; hsa-miR-570-5p
