# Supplementary material for: A comparison between SOLiD 5500XLand Ion Torrent PGM-derived miRNA expression profiles in two breast cell lines
Source: Genet Mol Biol. 2020 Apr 27;43(2):e20180351. doi: 10.1590/1678-4685-GMB-2018-0351 (PMC7201575; doi:10.1590/1678-4685-GMB-2018-0351)
Supplement: Table S2 - [file 1415-4757-GMB-43-2-e20180351-suppl4.pdf]

Supplementary Material to "A comparison between SOLiD 5500XL- and Ion Torrent PGM-derived miRNA expression profiles in two breast cell lines"

Table S2 – –Size factor used for normalizing the number of reads (sequencing depth) for each platform.

**Platform Cell line**	**Total Reads**	**1^6/total Factor**	**3*factor 3 reads**	**1.62/factor Reads = 3pgm C5.2 reads**
SOLiD - C5.2	8124149	0.12	0.36	13.5
SOLiD - HB4a	6833704	0.15	0.45	10.8
PGM - HB4a	3267637	0.31	0.93	5.23
PGM – C5.2	1852673	0.54	1.62	3
