# Supplementary material for: A comparison between SOLiD 5500XLand Ion Torrent PGM-derived miRNA expression profiles in two breast cell lines
Source: Genet Mol Biol. 2020 Apr 27;43(2):e20180351. doi: 10.1590/1678-4685-GMB-2018-0351 (PMC7201575; doi:10.1590/1678-4685-GMB-2018-0351)
Supplement: Table S3 - [file 1415-4757-GMB-43-2-e20180351-suppl5.pdf]

Supplementary Material to "A comparison between SOLiD 5500XL- and Ion Torrent PGM-derived miRNA expression profiles in two breast cell lines"

Table S3 - List of miRNAs identified for both cell lines in PGM and SOLiD.

miRNAs	miRNAs C5.2 PGM	miRNAs HB4a PGM	miRNAs C5.2 SOLiD	miRNAs HB4a SOLiD
hsa-let-7a-2-3p	33.8868097244093	6.22035461867352	17.675601113982	7.83516525659657
hsa-let-7a-3p	24.8503271312335	8.70849646614293	78.5582271732533	23.5054957697897
hsa-let-7b-3p	0	3.73221277120411	5.891867037994	1.04468870087954
hsa-let-7c-5p/let-7a-5p/let-7e-5p/let-7f-5p/let-7b-5p	3953.46113451442	2422.20608851147	4700.40059253299	3906.61339693905
hsa-let-7d-3p	9.03648259317581	2.48814184746941	10.4744302897671	5.74578785483749
hsa-let-7d-5p	10513.9474971601	6689.3693569215	3365.56538248079	2033.48655626203
hsa-let-7f-1-3p	11.2956032414698	6.22035461867352	17.675601113982	15.6703305131931
hsa-let-7f-2-3p	0	3.73221277120411	10.4744302897671	2.61172175219886
hsa-let-7g-3p	196.543496401574	200.295418721287	88.37800556991	92.9772943782793
hsa-let-7g-5p	5616.17393165877	4497.31638930096	4860.79030634505	7408.40992228728
hsa-let-7i-3p	33.8868097244093	19.9051347797553	51.7174995557251	18.8043966158318
hsa-let-7i-5p	3092.73616751442	2137.31384697622	583.294836761406	250.202943860651
hsa-miR-100-3p	169.434048622046	14.9288510848164	236.983985305981	17.2373635645125
hsa-miR-101-3p	3061.10847843831	1659.5906122621	5239.17910056289	2713.57890053461
hsa-miR-101-5p	27.1094477795274	3.73221277120411	18.3302530070924	7.83516525659657

hsa-miR-103a-2-5p	196.543496401574	113.210454059858	87.7233536767995	70.5164873093692
hsa-miR-103a-3p	28385.8509458135	24001.8603316136	367767.06725212	129542.443597764
hsa-miR-106b-3p	1059.52758404986	998.988951758967	798.675309594742	320.71943117002
hsa-miR-106b-5p	1753.07762307611	2048.98481139106	2563.6168134205	2085.72099130601
hsa-miR-107	1863.77453484251	2099.99171926418	10989.641329645	7167.08683238411
hsa-miR-10b-3p	0	0	0	6.79047655571703
hsa-miR-10b-5p/miR-10a-5p	323.054252706035	430.448539612208	305.722434082578	453.917240532161
hsa-miR-1180-3p	2.25912064829395	17.4169929322859	6.54651893110444	5.22344350439772
hsa-miR-1180-5p	6.77736194488186	21.14920570349	14.4023416484298	75.2175864633271
hsa-miR-1185-2-3p/miR-1185-1-3p	0	0	4.58256325177311	0
hsa-miR-1185-5p	11.2956032414698	0	5.23721514488355	0
hsa-miR-1248	2.25912064829395	0	10.4744302897671	4.17875480351817
hsa-miR-1249	4.51824129658791	0	111.945473721886	80.4410299677248
hsa-miR-1255a	4.51824129658791	11.1966383136123	4.58256325177311	1.04468870087954
hsa-miR-1256	2.25912064829395	23.6373475509594	4.58256325177311	15.1479861627534
hsa-miR-125a-3p	92.6239465800521	43.5424823307146	98.8524358596771	34.4747271290249
hsa-miR-125a-5p	1940.58463688451	1425.70527859997	2453.63529537795	1956.17959239694
hsa-miR-125b-1-3p	22.5912064829395	13.6847801610817	15.7116454346507	0
hsa-miR-125b-2-3p	4.51824129658791	0	4.58256325177311	1.04468870087954
hsa-miR-125b-5p	1027.89989497375	268.719319526696	1852.01020560945	760.011029889868
hsa-miR-1260b	42.9232923175851	223.932766272247	15.7116454346507	92.4549500278396

hsa-miR-126-3p	13852.9278153385	8653.7573454986	18674.5999028685	6759.13589469064
hsa-miR-126-5p	485.7109393832	251.30232659441	973.467365055231	627.335564878166
hsa-miR-1270	15.8138445380577	0	8.51047461043578	0
hsa-miR-1271-3p	22.5912064829395	22.3932766272247	16.3662973277611	19.8490853167113
hsa-miR-1271-5p	47.441533614173	62.2035461867352	70.0477525628175	180.208800901721
hsa-miR-1273c	6.77736194488186	7.46442554240822	28.8046832968596	25.5948731715488
hsa-miR-1273d	2.25912064829395	8.70849646614293	0	2.61172175219886
hsa-miR-1277-3p	2.25912064829395	1.2440709237347	11.783734075988	2.08937740175909
hsa-miR-1278	4.51824129658791	6.22035461867352	1.30930378622089	5.22344350439772
hsa-miR-128-1-5p	2.25912064829395	3.73221277120411	6.54651893110444	10.9692313592352
hsa-miR-128-3p	280.13096038845	498.872440417616	266.443320495951	455.484273583481
hsa-miR-1285-3p	15.8138445380577	69.6679717291434	54.3361071281669	149.912828576214
hsa-miR-1285-5p	6.77736194488186	4.97628369493882	3.27325946555222	4.70109915395794
hsa-miR-1286	0	12.440709237347	3.92791135866267	2.61172175219886
hsa-miR-1287-3p	0	1.2440709237347	1.30930378622089	6.79047655571703
hsa-miR-1287-5p	9.03648259317581	4.97628369493882	5.23721514488355	5.74578785483749
hsa-miR-129-2-3p/miR-129-1-3p	0	23.6373475509594	2.61860757244178	16.1926748636329
hsa-miR-129-5p	0	4.97628369493882	0	0.522344350439772
hsa-miR-1296-3p	6.77736194488186	3.73221277120411	1.96395567933133	5.74578785483749
hsa-miR-1296-5p	352.422821133857	161.729220085512	42.5523730521789	25.072528821109
hsa-miR-1301-3p	13.5547238897637	36.0780567883064	15.7116454346507	16.1926748636329

hsa-miR-1304-3p	2.25912064829395	4.97628369493882	1.96395567933133	3.13406610263863
hsa-miR-1305	2.25912064829395	4.97628369493882	2.61860757244178	0
hsa-miR-1306-5p	15.8138445380577	34.8339858645717	25.5314238313073	37.0864488812238
hsa-miR-1307-3p	158.138445380577	206.515773339961	62.8465817386027	72.6058647111282
hsa-miR-1307-5p	2934.59772213384	2631.2100036989	525.685470167687	536.969992252085
hsa-miR-130a-3p	22963.961389908	34515.5037080956	53680.8005831633	67864.022697836
hsa-miR-130b-3p	2909.74739500261	2224.39881163765	8179.875404415	4554.32039148437
hsa-miR-130b-5p	171.69316927034	90.8171774326334	222.581643657551	81.4857186686044
hsa-miR-132-3p	22.5912064829395	26.1254893984288	54.9907590212773	87.2315065234419
hsa-miR-132-5p	88.1057052834642	106.990099441185	66.1198412041549	91.41026132696
hsa-miR-1343-3p	2.25912064829395	7.46442554240822	3.92791135866267	4.17875480351817
hsa-miR-134-5p	2.25912064829395	0	8.51047461043578	0.522344350439772
hsa-miR-135a-5p/miR-135b-5p	442.787647065615	1042.53143408968	381.662053683389	719.268170555565
hsa-miR-135b-3p	0	7.46442554240822	0.654651893110444	16.1926748636329
hsa-miR-137	124.251635656167	29.8577021696329	82.486138531916	24.5501844706693
hsa-miR-138-1-3p	15.8138445380577	41.0543404832452	17.0209492208716	25.072528821109
hsa-miR-138-5p	370.495786320208	882.046284927905	600.970437875388	2622.69098355809
hsa-miR-139-5p	13.5547238897637	3.73221277120411	3.27325946555222	5.22344350439772
hsa-miR-140-3p	1210.88866748556	666.822015121801	1835.64390828169	1413.98615664046
hsa-miR-140-5p	42.9232923175851	26.1254893984288	163.008321384501	56.9355341979351
hsa-miR-141-3p	13.5547238897637	46.030624178184	9.16512650354622	42.8322367360613

hsa-miR-141-5p	4.51824129658791	28.6136312458982	21.6035124726447	31.8630053768261
hsa-miR-142-3p	2.25912064829395	2.48814184746941	3.92791135866267	5.22344350439772
hsa-miR-142-5p	2.25912064829395	0	34.6965503348536	22.9831514193499
hsa-miR-143-3p	0	2.48814184746941	4.58256325177311	7.3128209061568
hsa-miR-145-5p	6.77736194488186	3.73221277120411	34.6965503348536	23.5054957697897
hsa-miR-146b-5p	36.1459303727032	32.3458440171023	13.7476897553193	14.6256418123136
hsa-miR-147b	11.2956032414698	113.210454059858	4.58256325177311	36.564104530784
hsa-miR-148a-3p	1003.04956784252	3994.71173611213	1507.66330983335	10044.6818589568
hsa-miR-148a-5p	0	11.1966383136123	2.61860757244178	7.3128209061568
hsa-miR-148b-3p	661.922349950128	582.225192307842	1034.3499911145	536.447647901645
hsa-miR-148b-5p	29.3685684278214	53.4950497205923	15.0569935415402	12.0139200601147
hsa-miR-149-3p	2.25912064829395	13.6847801610817	1.96395567933133	8.35750960703635
hsa-miR-149-5p	205.57997899475	475.235092866657	192.467656574471	508.241052977898
hsa-miR-150-5p	0	1.2440709237347	74.6303158145907	43.8769254369408
hsa-miR-151a-3p	856.206725703408	338.38729125584	481.169141436177	397.504050684666
hsa-miR-151a-5p	2385.63140459841	1811.36726495773	1772.14267464997	1685.60521886914
hsa-miR-151b	63.2553781522307	27.3695603221635	83.7954423181369	34.4747271290249
hsa-miR-152-3p	323.054252706035	631.98802925723	149.260631629181	314.973643315182
hsa-miR-152-5p	6.77736194488186	17.4169929322859	81.1768347456951	188.043966158318
hsa-miR-153-5p	9.03648259317581	0	0	0
hsa-miR-1537-3p	4.51824129658791	7.46442554240822	1.96395567933133	8.35750960703635
hsa-miR-154-3p	13.5547238897637	1.2440709237347	19.6395567933133	2.61172175219886

hsa-miR-154-5p	6.77736194488186	0	0	1.04468870087954
hsa-miR-155-3p	24.8503271312335	0	127.002467263426	5.22344350439772
hsa-miR-155-5p	115.215153062992	8.70849646614293	130.275726728978	15.6703305131931
hsa-miR-15a-3p	395.346113451442	389.394199128962	780.34505658765	951.189062150824
hsa-miR-15a-5p	1317.06733795537	1525.23095249875	930.260340109941	1304.81618739855
hsa-miR-15b-3p	1353.21326832808	1191.81994493785	1127.31055993619	868.1363104309
hsa-miR-15b-5p	3515.19172874539	2577.71495397831	6682.03187297831	3838.70863138188
hsa-miR-16-1-3p	115.215153062992	104.501957593715	3.92791135866267	2.08937740175909
hsa-miR-16-2-3p	1524.90643759842	840.99194444466	928.951036323721	911.490891517401
hsa-miR-16-5p	6991.97840646978	13683.536090158	6345.54079991954	10490.2415898819
hsa-miR-17-3p	13911.6649521942	13793.0143314467	5180.91508207606	9114.90891517401
hsa-miR-17-5p/miR-106a-5p	15504.3450092414	17969.3604224241	39158.6576382943	52637.1625381662
hsa-miR-181a-2-3p	13.5547238897637	44.7865532544493	24.8767719381969	48.5780245908988
hsa-miR-181a-3p	526.375111052491	278.671886916574	1780.65314926041	366.16338965828
hsa-miR-181a-5p	2024.17210087138	969.131249589335	2335.79795461807	1248.40299755105
hsa-miR-181b-3p	2.25912064829395	2.48814184746941	5.891867037994	43.8769254369408
hsa-miR-181b-5p/miR-181d-5p	668.69971189501	467.770667324249	411.121388873359	428.322367360613
hsa-miR-181c-5p	33.8868097244093	17.4169929322859	27.4953795106387	15.6703305131931
hsa-miR-182-3p	56.4780162073488	136.847801610817	69.3931006697071	101.857148335755
hsa-miR-182-5p	2618.32083137269	3429.90353673658	1096.54192095999	2462.85361232352
hsa-miR-183-3p	4.51824129658791	11.1966383136123	9.81977839665667	21.9384627184704

hsa-miR-183-5p	9.03648259317581	23.6373475509594	2.61860757244178	11.491575709675
hsa-miR-185-3p	0	7.46442554240822	7.20117082421489	7.3128209061568
hsa-miR-185-5p	5381.2253842362	12980.6360182479	5957.98687919815	8703.82391137791
hsa-miR-186-3p	13.5547238897637	21.14920570349	55.6454109143878	98.7230822331168
hsa-miR-186-5p	271.094477795274	260.010823060553	607.516956806492	436.679876967649
hsa-miR-187-3p	2.25912064829395	6.22035461867352	11.783734075988	17.2373635645125
hsa-miR-188-3p	2.25912064829395	8.70849646614293	35.351202227964	33.9523827785852
hsa-miR-188-5p	192.025255104986	309.773660009941	254.004934526852	225.130415039542
hsa-miR-18a-3p	33.8868097244093	46.030624178184	20.9488605795342	31.3406610263863
hsa-miR-18b-5p/miR-18a-5p	1893.14310327033	4891.68687212486	1478.20397464338	6675.56079862028
hsa-miR-190a-3p	4.51824129658791	1.2440709237347	7.20117082421489	3.6564104530784
hsa-miR-190a-5p	36.1459303727032	2.48814184746941	65.4651893110444	13.0586087609943
hsa-miR-1910-3p	4.51824129658791	28.6136312458982	5.891867037994	12.5362644105545
hsa-miR-1910-5p	0	9.95256738987763	0	6.79047655571703
hsa-miR-191-3p	0	7.46442554240822	3.27325946555222	10.4468870087954
hsa-miR-1914-3p	2.25912064829395	4.97628369493882	1.30930378622089	4.17875480351817
hsa-miR-191-5p	3540.04205587662	4601.81834689467	3629.3900954043	5181.65595636253
hsa-miR-192-3p	54.2188955590549	59.7154043392658	32.7325946555222	60.0696003005737
hsa-miR-192-5p	1870.55189678739	3222.14369247288	2401.91779582222	5130.46621001944
hsa-miR-193a-3p	106.178670469816	95.7934611275722	59.5733222730504	66.337732505851
hsa-miR-193a-5p	15.8138445380577	22.3932766272247	35.351202227964	59.547255950134
hsa-miR-193b-3p	74.5509813937005	406.811192061248	130.930378622089	598.606625603978

hsa-miR-193b-5p	0	16.1729220085512	15.7116454346507	55.3685011466158
hsa-miR-194-3p	0	2.48814184746941	0.654651893110444	5.74578785483749
hsa-miR-194-5p	83.5874639868763	154.264794543103	144.678068377408	170.284258243366
hsa-miR-195-3p	0	3.73221277120411	2.61860757244178	7.83516525659657
hsa-miR-195-5p	47.441533614173	329.678794789697	38.6244616935162	629.424942279925
hsa-miR-196a-3p	83.5874639868763	44.7865532544493	187.230441429587	66.337732505851
hsa-miR-196b-3p	56.4780162073488	47.2746951019188	92.9605688216831	143.644696370937
hsa-miR-196b-5p/miR-196a-5p	517.338628459315	574.760766765433	381.662053683389	406.383904642142
hsa-miR-1972	0	1.2440709237347	4.58256325177311	1.04468870087954
hsa-miR-197-3p	243.985030015747	431.692610535942	54.9907590212773	153.046894678853
hsa-miR-199a-5p	2.25912064829395	0	52.3721514488356	32.3853497272658
hsa-miR-199b-3p/miR-199a-3p	2.25912064829395	3.73221277120411	60.8826260592713	64.2483551040919
hsa-miR-199b-5p	24.8503271312335	65.9357589579393	98.1977839665667	112.826379694991
hsa-miR-19a-5p	4.51824129658791	2.48814184746941	4.58256325177311	1.56703305131931
hsa-miR-19b-1-5p	323.054252706035	344.607645874513	284.118921609933	330.643973828375
hsa-miR-19b-3p/miR-19a-3p	119582.033276144	84177.5709126613	181295.367366648	114583.02374552
hsa-miR-200a-3p	0	7.46442554240822	8.51047461043578	15.6703305131931
hsa-miR-200b-3p	2.25912064829395	8.70849646614293	5.891867037994	8.87985395747612
hsa-miR-200c-3p	1122.78296220209	2786.71886916574	153.843194880954	707.254250495451
hsa-miR-200c-5p	9.03648259317581	63.4476171104699	6.54651893110444	13.0586087609943

hsa-miR-203a	250.762391960629	559.831915680617	189.194397108918	428.322367360613
hsa-miR-204-5p	0	4.97628369493882	0.654651893110444	1.56703305131931
hsa-miR-205-3p	6.77736194488186	3.73221277120411	0	2.61172175219886
hsa-miR-205-5p	1393.87743999737	4437.60098496169	1239.91068555118	4238.30205946831
hsa-miR-20a-3p	2695.13093341469	1662.07875410956	374.460882859174	45.9663028386999
hsa-miR-20a-5p	1922.51167169815	2280.38200320571	3700.74715175334	3702.3767559171
hsa-miR-20b-3p	0	11.1966383136123	0.654651893110444	3.6564104530784
hsa-miR-20b-5p	4.51824129658791	57.2272624917964	43.8616768383998	309.227855460345
hsa-miR-210-3p	2747.09070832545	4324.39053090183	6212.64646561812	4097.79142920001
hsa-miR-210-5p	6.77736194488186	33.589914940837	11.783734075988	27.1619062228681
hsa-miR-2110	0	9.95256738987763	0.654651893110444	3.6564104530784
hsa-miR-2116-5p	0	4.97628369493882	2.61860757244178	6.79047655571703
hsa-miR-21-3p	5071.72585541992	1634.7091937874	3930.52996623511	1745.15247481928
hsa-miR-215-5p	15.8138445380577	11.1966383136123	10.4744302897671	14.6256418123136
hsa-miR-21-5p	913120.752195878	447494.799409221	466698.715990863	71900.6998380345
hsa-miR-216a-3p	11.2956032414698	3.73221277120411	7.85582271732533	3.6564104530784
hsa-miR-216a-5p	29.3685684278214	6.22035461867352	15.0569935415402	0
hsa-miR-217	18.0729651863516	3.73221277120411	7.85582271732533	3.6564104530784
hsa-miR-218-2-3p	13.5547238897637	4.97628369493882	5.891867037994	6.26813220527726
hsa-miR-218-5p	661.922349950128	522.509787968576	1052.68024412159	740.684288923596
hsa-miR-219a-5p	2.25912064829395	6.22035461867352	5.891867037994	10.9692313592352
hsa-miR-219b-5p	6.77736194488186	1.2440709237347	0.654651893110444	2.08937740175909

hsa-miR-221-3p	15088.6668099553	15483.7067168021	26207.6792368904	12758.2607594914
hsa-miR-221-5p	92.6239465800521	80.8646100427558	100.816391539008	72.0835203606885
hsa-miR-222-3p	8858.01206196059	8433.55679199756	9918.63083251634	4220.02000720291
hsa-miR-222-5p	564.780162073488	323.458440171023	968.884801803458	182.29817830348
hsa-miR-223-3p	0	0	36.0058541210744	9.40219830791589
hsa-miR-22-3p	1183.77921970603	1953.19135026349	411.121388873359	1015.95976160536
hsa-miR-224-3p	2.25912064829395	2.48814184746941	4.58256325177311	6.79047655571703
hsa-miR-224-5p	1280.92140758267	315.994014628615	1600.62387865504	309.227855460345
hsa-miR-22-5p	304.981287519684	569.784483070495	365.295756355628	608.008823911894
hsa-miR-2276-3p	9.03648259317581	6.22035461867352	3.27325946555222	2.61172175219886
hsa-miR-2277-3p	11.2956032414698	6.22035461867352	5.23721514488355	10.4468870087954
hsa-miR-2277-5p	6.77736194488186	12.440709237347	9.81977839665667	8.87985395747612
hsa-miR-2278	6.77736194488186	14.9288510848164	5.23721514488355	6.26813220527726
hsa-miR-2355-3p	9.03648259317581	2.48814184746941	11.783734075988	13.5809531114341
hsa-miR-2355-5p	13.5547238897637	14.9288510848164	19.6395567933133	32.9076940777056
hsa-miR-23a-3p/miR-23b-3p	45995.6963992649	30423.7544399322	61252.5043788787	34752.6143234589
hsa-miR-23a-5p	6.77736194488186	6.22035461867352	16.3662973277611	5.74578785483749
hsa-miR-23c	2.25912064829395	6.22035461867352	5.891867037994	11.491575709675
hsa-miR-24-1-5p	22.5912064829395	13.6847801610817	18.3302530070924	19.8490853167113
hsa-miR-24-2-5p	964.644516821518	945.493902038375	817.005562601835	668.078424212468
hsa-miR-24-3p	28040.2054866245	24916.2524605587	17747.6128222241	9612.18073679268
hsa-miR-25-3p	1511.35171370865	1504.08174679526	2619.916876228	3037.43239780727

hsa-miR-25-5p	33.8868097244093	36.0780567883064	2.61860757244178	1.04468870087954
hsa-miR-2682-3p	15.8138445380577	9.95256738987763	15.7116454346507	6.79047655571703
hsa-miR-2682-5p	2.25912064829395	3.73221277120411	7.20117082421489	1.04468870087954
hsa-miR-26a-1-3p	20.3320858346456	31.1017730933676	30.7686389761909	44.3992697873806
hsa-miR-26a-2-3p	36.1459303727032	11.1966383136123	45.8256325177311	12.0139200601147
hsa-miR-26a-5p	7199.81750611283	6919.52247781242	5595.30973041497	6218.50949198548
hsa-miR-26b-3p	85.8465846351702	44.7865532544493	105.398954790782	33.9523827785852
hsa-miR-26b-5p	1400.65480194225	719.072993918659	1744.64729513933	618.978055271129
hsa-miR-27a-5p	144.583721490813	77.1323972715517	484.442400901729	76.7846195146464
hsa-miR-27b-3p/miR-27a-3p	20445.0418670603	12445.685521042	9341.88251468604	3890.42072207542
hsa-miR-27b-5p	33.8868097244093	27.3695603221635	76.594271493922	15.6703305131931
hsa-miR-28-3p	264.317115850392	133.115588839613	409.157433194028	200.580230568872
hsa-miR-28-5p	420.196440582675	179.146213017797	100.161739645898	45.9663028386999
hsa-miR-296-3p	1260.58932174803	837.259731673456	1156.76989512616	727.103335812162
hsa-miR-296-5p	286.908322333332	191.586922255144	590.496007585621	452.350207480842
hsa-miR-299-3p	0	0	5.891867037994	0
hsa-miR-299-5p	9.03648259317581	0	18.9849049002029	1.56703305131931
hsa-miR-29a-5p	291.42656362992	121.918950526001	333.872465486327	126.407332806425
hsa-miR-29b-1-5p	115.215153062992	58.4713334155311	150.569935415402	144.689385071817
hsa-miR-29b-2-5p	15.8138445380577	21.14920570349	19.6395567933133	33.9523827785852
hsa-miR-29b-3p	32562.965024509	44200.5958493703	50978.3975684034	140499.661036939
hsa-miR-29c-3p/miR-29a-3p	5308.93352349079	4952.64634738786	34210.7986301656	69484.3348729002

hsa-miR-29c-5p	106.178670469816	133.115588839613	121.110600225432	100.812459634876
hsa-miR-301a-3p	9870.09811239628	7209.39100304261	26214.8804077146	16077.2367621857
hsa-miR-301a-5p	117.474273711286	121.918950526001	101.471043432119	146.778762473576
hsa-miR-301b	225.912064829395	391.882340976432	1527.95751851978	1079.16342800857
hsa-miR-3065-3p	40.6641716692912	334.655078484635	18.9849049002029	167.150192140727
hsa-miR-3065-5p	142.324600842519	1545.1360872785	53.026803341946	887.985395747612
hsa-miR-3074-3p	6.77736194488186	9.95256738987763	8.51047461043578	4.70109915395794
hsa-miR-30b-3p	33.8868097244093	47.2746951019188	4.58256325177311	9.40219830791589
hsa-miR-30b-5p	81545.2189208185	86119.5656246112	82889.404098072	154964.943133668
hsa-miR-30c-1-3p	29.3685684278214	11.1966383136123	4.58256325177311	1.56703305131931
hsa-miR-30c-2-3p	24.8503271312335	17.4169929322859	1.30930378622089	14.1032974618738
hsa-miR-30c-5p	11733.8726472388	12079.928669464	14169.9402263756	26949.8344165896
hsa-miR-30d-3p	38.4050510209972	37.3221277120411	15.0569935415402	21.4161183680306
hsa-miR-30e-3p/miR-30a-3p	135.547238897637	128.139305144675	150.569935415402	59.0249115996942
hsa-miR-30e-5p/miR-30a-5p/miR-30d-5p	7577.09065437792	6439.31110125083	9671.1724169206	4351.1284391633
hsa-miR-3130-5p	0	4.97628369493882	0.654651893110444	4.70109915395794
hsa-miR-31-3p	533.152472997373	237.617546433328	809.149739884509	263.783896972085
hsa-miR-3144-3p	0	0	4.58256325177311	0
hsa-miR-3158-5p	4.51824129658791	0	0	0.522344350439772
hsa-miR-31-5p	42841.9639742465	15778.5515257273	37937.0772057503	10594.7104599699
hsa-miR-3176	11.2956032414698	7.46442554240822	9.81977839665667	13.0586087609943

hsa-miR-3177-3p	6.77736194488186	12.440709237347	3.27325946555222	11.491575709675
hsa-miR-3179	2.25912064829395	3.73221277120411	5.23721514488355	3.13406610263863
hsa-miR-3182	2.25912064829395	9.95256738987763	5.891867037994	4.70109915395794
hsa-miR-3187-3p	0	4.97628369493882	0	0
hsa-miR-3194-5p	29.3685684278214	130.627446992144	44.5163287315102	61.636633351893
hsa-miR-3200-3p	74.5509813937005	131.871517915879	73.9756639214802	174.463013046884
hsa-miR-320a	304.981287519684	629.49988740976	172.173447888047	1137.66599525782
hsa-miR-320b	4.51824129658791	12.440709237347	2.61860757244178	15.1479861627534
hsa-miR-320c	4.51824129658791	7.46442554240822	1.30930378622089	3.6564104530784
hsa-miR-323b-3p	20.3320858346456	0	0.654651893110444	1.56703305131931
hsa-miR-32-3p	101.660429173228	93.3053192801028	101.471043432119	56.4131898474953
hsa-miR-324-3p	51.9597749107609	73.4001845003475	5.891867037994	21.9384627184704
hsa-miR-324-5p	237.207668070865	486.431731180269	534.850596671233	774.636671702181
hsa-miR-32-5p	234.948547422571	262.498964908023	262.515409137288	351.015403495526
hsa-miR-326	24.8503271312335	92.0612483563681	43.8616768383998	95.0666717800384
hsa-miR-328-3p	38.4050510209972	47.2746951019188	24.2221200450864	43.8769254369408
hsa-miR-329-3p	11.2956032414698	1.2440709237347	8.51047461043578	2.08937740175909
hsa-miR-330-3p	33.8868097244093	29.8577021696329	17.0209492208716	28.7289392741874
hsa-miR-330-5p	11.2956032414698	8.70849646614293	0	1.04468870087954
hsa-miR-331-3p	431.492043824145	496.384298570147	482.478445222398	319.67474246914
hsa-miR-331-5p	101.660429173228	164.217361932981	70.702404455928	66.337732505851
hsa-miR-335-3p	72.2918607454065	140.580014382022	39.2791135866267	65.8153881554112

hsa-miR-335-5p	214.616461587926	641.940596647107	223.890947443772	254.381698664169
hsa-miR-339-3p	309.499528816272	415.519688527391	97.5431320734562	241.323089903174
hsa-miR-339-5p	1079.85966988451	1375.94244165058	838.609075074479	1269.81911591908
hsa-miR-33a-3p	79.0692226902884	48.5187660256535	68.7384487765967	119.094511900268
hsa-miR-33b-3p	128.769876952755	80.8646100427558	282.809617823712	108.647624891472
hsa-miR-33b-5p	29.3685684278214	2.48814184746941	57.6093665937191	29.2512836246272
hsa-miR-340-3p	589.630489204722	422.984114069799	1135.16638265351	678.002966870823
hsa-miR-340-5p	70.0327400971125	8.70849646614293	53.026803341946	1.56703305131931
hsa-miR-342-3p	562.521041425194	641.940596647107	673.636798010647	829.482828498357
hsa-miR-342-5p	4.51824129658791	3.73221277120411	3.27325946555222	8.35750960703635
hsa-miR-345-5p	646.108505412071	607.106610782536	564.309931861203	529.657171345928
hsa-miR-34a-3p	0	14.9288510848164	5.891867037994	45.9663028386999
hsa-miR-34a-5p	320.795132057741	3635.17523915281	299.830567044584	5145.61419618219
hsa-miR-34b-3p	0	6.22035461867352	0	6.26813220527726
hsa-miR-34b-5p	29.3685684278214	250.058255670676	24.2221200450864	421.009546454456
hsa-miR-34c-5p	20.3320858346456	171.681787475389	20.9488605795342	121.706233652467
hsa-miR-3605-3p	6.77736194488186	2.48814184746941	3.92791135866267	3.13406610263863
hsa-miR-3607-5p	2.25912064829395	3.73221277120411	6007.74042307455	9792.91188204484
hsa-miR-3613-3p	4.51824129658791	4.97628369493882	0.654651893110444	1.56703305131931
hsa-miR-3613-5p	45.1824129658791	19.9051347797553	0	1.04468870087954
hsa-miR-361-3p	47.441533614173	19.9051347797553	74.6303158145907	25.5948731715488
hsa-miR-3615	13.5547238897637	13.6847801610817	6.54651893110444	4.17875480351817

hsa-miR-361-5p	88.1057052834642	26.1254893984288	102.78034721834	43.354581086501
hsa-miR-3616-5p	6.77736194488186	0	0.654651893110444	0
hsa-miR-3617-5p	6.77736194488186	21.14920570349	0.654651893110444	5.74578785483749
hsa-miR-3618	2.25912064829395	1.2440709237347	4.58256325177311	5.22344350439772
hsa-miR-3619-5p	2.25912064829395	14.9288510848164	6.54651893110444	3.13406610263863
hsa-miR-362-3p	56.4780162073488	28.6136312458982	77.2489233870324	28.7289392741874
hsa-miR-362-5p	110.696911766404	120.674879602266	127.657119156537	87.2315065234419
hsa-miR-363-3p	4.51824129658791	41.0543404832452	1.30930378622089	45.4439584882601
hsa-miR-3651	9.03648259317581	2.48814184746941	3.92791135866267	3.13406610263863
hsa-miR-365b-3p/miR-365a-3p	20.3320858346456	48.5187660256535	25.5314238313073	19.8490853167113
hsa-miR-3677-3p	15.8138445380577	28.6136312458982	5.891867037994	14.1032974618738
hsa-miR-3677-5p	2.25912064829395	4.97628369493882	6.54651893110444	8.87985395747612
hsa-miR-3687	13.5547238897637	33.589914940837	0.654651893110444	2.61172175219886
hsa-miR-369-5p	6.77736194488186	0	2.61860757244178	0
hsa-miR-374a-3p	92.6239465800521	22.3932766272247	149.260631629181	17.7597079149522
hsa-miR-374a-5p	530.893352349079	128.139305144675	621.919298454922	48.5780245908988
hsa-miR-374b-3p	79.0692226902884	103.25788666998	117.18268886677	53.2791237448567
hsa-miR-374b-5p	786.173985606296	400.590837442575	925.677776858168	283.632982288796
hsa-miR-376a-3p	15.8138445380577	7.46442554240822	25.5314238313073	6.26813220527726
hsa-miR-376a-5p	38.4050510209972	18.6610638560206	53.026803341946	9.92454265835566
hsa-miR-376b-3p	33.8868097244093	11.1966383136123	20.9488605795342	6.26813220527726

hsa-miR-376b-5p	9.03648259317581	0	15.0569935415402	1.56703305131931
hsa-miR-376c-3p	345.645459188975	74.6442554240822	202.287434971127	36.0417601803442
hsa-miR-376c-5p	11.2956032414698	0	17.675601113982	1.56703305131931
hsa-miR-378a-3p	4845.81379059053	4252.23441732522	2327.28748000763	3160.70566451106
hsa-miR-378a-5p	201.061737698162	185.366567636471	456.29236949798	433.54581086501
hsa-miR-378c	264.317115850392	190.34285133141	233.056073947318	181.77583395304
hsa-miR-378d	79.0692226902884	90.8171774326334	56.3000628074982	40.2205149838624
hsa-miR-378f	22.5912064829395	13.6847801610817	11.783734075988	10.4468870087954
hsa-miR-378g	2.25912064829395	6.22035461867352	1.30930378622089	1.56703305131931
hsa-miR-378i	24.8503271312335	17.4169929322859	25.5314238313073	25.072528821109
hsa-miR-379-5p	42.9232923175851	9.95256738987763	25.5314238313073	10.9692313592352
hsa-miR-381-3p	22.5912064829395	3.73221277120411	15.0569935415402	2.61172175219886
hsa-miR-381-5p	2.25912064829395	1.2440709237347	5.23721514488355	0.522344350439772
hsa-miR-382-5p	4.51824129658791	0	5.23721514488355	0
hsa-miR-3912-3p	9.03648259317581	12.440709237347	17.675601113982	8.87985395747612
hsa-miR-3913-3p	9.03648259317581	2.48814184746941	5.23721514488355	1.04468870087954
hsa-miR-3913-5p	4.51824129658791	2.48814184746941	9.81977839665667	5.74578785483749
hsa-miR-3926	4.51824129658791	0	0.654651893110444	0
hsa-miR-3928-5p	0	0	5.23721514488355	1.56703305131931
hsa-miR-3929	2.25912064829395	4.97628369493882	1.30930378622089	2.61172175219886
hsa-miR-3934-3p	2.25912064829395	1.2440709237347	1.30930378622089	7.3128209061568
hsa-miR-3934-5p	6.77736194488186	21.14920570349	0	11.491575709675

hsa-miR-3935	2.25912064829395	4.97628369493882	4.58256325177311	5.22344350439772
hsa-miR-409-3p	33.8868097244093	2.48814184746941	23.567468151976	3.13406610263863
hsa-miR-409-5p	9.03648259317581	1.2440709237347	1.96395567933133	1.04468870087954
hsa-miR-421	449.565009010497	998.988951758967	406.538825621586	1033.71946952031
hsa-miR-422a	4.51824129658791	7.46442554240822	1.30930378622089	2.08937740175909
hsa-miR-423-3p	67.7736194488186	189.098780407675	101.471043432119	173.940668696444
hsa-miR-423-5p	289.167442981626	187.85470948394	108.017562363223	107.602936190593
hsa-miR-424-3p	94.883067228346	92.0612483563681	21.6035124726447	1.04468870087954
hsa-miR-424-5p	1391.61831934908	1584.94635683801	6031.96254311963	7583.91762403504
hsa-miR-425-3p	49.700654262467	106.990099441185	29.45933518997	92.9772943782793
hsa-miR-425-5p	1888.62486197374	3213.43519600674	2386.86080228068	8257.74183610235
hsa-miR-4256	0	0	10.4744302897671	10.4468870087954
hsa-miR-4284	4.51824129658791	44.7865532544493	824.86138531916	861.868178225623
hsa-miR-4286	67.7736194488186	115.698595907327	51.7174995557251	87.2315065234419
hsa-miR-429	6.77736194488186	12.440709237347	0	5.22344350439772
hsa-miR-4326	2.25912064829395	2.48814184746941	17.0209492208716	10.4468870087954
hsa-miR-4429	2.25912064829395	8.70849646614293	0	0
hsa-miR-4454	49.700654262467	38.5661986357758	30.7686389761909	8.87985395747612
hsa-miR-4455	0	14.9288510848164	0	0
hsa-miR-4461	0	6.22035461867352	6.54651893110444	9.92454265835566
hsa-miR-4484	4.51824129658791	1.2440709237347	0	0.522344350439772
hsa-miR-4485	6.77736194488186	3.73221277120411	9.81977839665667	36.0417601803442

hsa-miR-449a	9.03648259317581	1.2440709237347	7.20117082421489	3.6564104530784
hsa-miR-449b-5p	2.25912064829395	6.22035461867352	11.1290821828776	3.6564104530784
hsa-miR-449c-5p	9.03648259317581	3.73221277120411	3.92791135866267	2.08937740175909
hsa-miR-4504	0	2.48814184746941	2.61860757244178	5.22344350439772
hsa-miR-450a-5p	13.5547238897637	12.440709237347	7.20117082421489	1.04468870087954
hsa-miR-450b-5p	24.8503271312335	14.9288510848164	6.54651893110444	0.522344350439772
hsa-miR-451a	2.25912064829395	3.73221277120411	30.1139870830804	28.7289392741874
hsa-miR-4521	72.2918607454065	119.430808678532	624.537906027364	477.945080652391
hsa-miR-4523	0	6.22035461867352	0	2.08937740175909
hsa-miR-4524a-5p	0	0	4.58256325177311	3.13406610263863
hsa-miR-452-5p	404.382596044618	217.712411653573	505.391261481263	497.271821618662
hsa-miR-454-3p	3463.23195383463	1700.64495274534	2396.02592878423	781.427148257898
hsa-miR-454-5p	160.397566028871	125.651163297205	439.926072170219	204.236641021951
hsa-miR-455-3p	948.83067228346	674.28644066421	889.017270843984	358.850568752123
hsa-miR-455-5p	293.685684278214	104.501957593715	238.947940985312	164.538470388528
hsa-miR-4664-5p	63.2553781522307	51.0069078731229	28.8046832968596	37.6087932316636
hsa-miR-4705	6.77736194488186	2.48814184746941	0.654651893110444	0
hsa-miR-4707-3p	15.8138445380577	19.9051347797553	17.675601113982	16.7150192140727
hsa-miR-4714-3p	0	0	5.891867037994	1.56703305131931
hsa-miR-4726-5p	0	6.22035461867352	0	4.70109915395794
hsa-miR-4731-3p	6.77736194488186	0	3.92791135866267	0.522344350439772
hsa-miR-4746-5p	6.77736194488186	3.73221277120411	14.4023416484298	5.74578785483749

hsa-miR-4761-5p	0	9.95256738987763	5.891867037994	3.6564104530784
hsa-miR-4762-5p	4.51824129658791	1.2440709237347	0	0
hsa-miR-4785	0	7.46442554240822	3.92791135866267	3.13406610263863
hsa-miR-483-3p	20.3320858346456	3.73221277120411	20.2942086864238	1.56703305131931
hsa-miR-483-5p	47.441533614173	1.2440709237347	33.3872465486327	1.56703305131931
hsa-miR-484	5162.09068135168	8962.28693458481	3631.35405108364	5982.4098455867
hsa-miR-485-3p	0	0	7.20117082421489	0
hsa-miR-486-5p	18.0729651863516	8.70849646614293	0.654651893110444	2.08937740175909
hsa-miR-487a-3p	4.51824129658791	0	1.96395567933133	0
hsa-miR-487b-3p	18.0729651863516	7.46442554240822	31.4232908693013	3.6564104530784
hsa-miR-491-3p	0	1.2440709237347	5.23721514488355	0
hsa-miR-491-5p	18.0729651863516	7.46442554240822	15.7116454346507	5.74578785483749
hsa-miR-494-3p	38.4050510209972	4.97628369493882	20.9488605795342	5.22344350439772
hsa-miR-495-3p	11.2956032414698	2.48814184746941	18.9849049002029	3.13406610263863
hsa-miR-497-3p	4.51824129658791	7.46442554240822	2.61860757244178	12.5362644105545
hsa-miR-497-5p	2.25912064829395	13.6847801610817	7.85582271732533	20.8937740175909
hsa-miR-5000-3p	4.51824129658791	0	0	0
hsa-miR-5008-5p	31.6276890761153	26.1254893984288	13.0930378622089	8.87985395747612
hsa-miR-500a-3p	13.5547238897637	14.9288510848164	15.7116454346507	2.61172175219886
hsa-miR-500a-5p	36.1459303727032	41.0543404832452	49.7535438763938	72.0835203606885
hsa-miR-500b-5p	27.1094477795274	22.3932766272247	32.0779427624118	53.8014680952965
hsa-miR-501-3p	4.51824129658791	0	1.30930378622089	0.522344350439772

hsa-miR-501-5p	6.77736194488186	8.70849646614293	2.61860757244178	15.1479861627534
hsa-miR-502-3p	20.3320858346456	16.1729220085512	7.85582271732533	3.6564104530784
hsa-miR-502-5p	108.43779111811	155.508865466838	60.2279741661609	132.153120661262
hsa-miR-503-5p	368.236665671914	475.235092866657	158.425758132728	190.655687910517
hsa-miR-504-5p	11.2956032414698	0	6.54651893110444	0
hsa-miR-505-3p	725.177728102359	379.441631739085	906.038220064855	207.370707124589
hsa-miR-5095	2.25912064829395	4.97628369493882	0.654651893110444	1.56703305131931
hsa-miR-5096	0	4.97628369493882	0	2.61172175219886
hsa-miR-519a-3p	0	0	3.27325946555222	12.5362644105545
hsa-miR-532-3p	282.390081036744	227.664979043451	49.7535438763938	57.4578785483749
hsa-miR-532-5p	980.458361359576	1268.9523422094	4712.18432660898	3839.23097573232
hsa-miR-542-3p	81.3283433385823	99.5256738987763	142.714112698077	334.822728631894
hsa-miR-542-5p	27.1094477795274	48.5187660256535	142.714112698077	150.957517277094
hsa-miR-543	4.51824129658791	0	1.30930378622089	0.522344350439772
hsa-miR-545-3p	20.3320858346456	93.3053192801028	54.9907590212773	229.30916984306
hsa-miR-545-5p	49.700654262467	102.013815746246	108.672214256334	373.476210564437
hsa-miR-548a-3p	33.8868097244093	3.73221277120411	6.54651893110444	0
hsa-miR-548ah-3p/miR-548aq-3p/miR-548am-3p	6.77736194488186	2.48814184746941	5.891867037994	2.08937740175909
hsa-miR-548ai/miR-570-5p	9.03648259317581	6.22035461867352	3.92791135866267	5.74578785483749
hsa-miR-548ao-5p	0	4.97628369493882	0	0
hsa-miR-548aq-5p	31.6276890761153	16.1729220085512	8.51047461043578	5.22344350439772

hsa-miR-548aw	9.03648259317581	4.97628369493882	7.20117082421489	3.13406610263863
hsa-miR-548b-3p	0	1.2440709237347	5.891867037994	0.522344350439772
hsa-miR-548d-3p	6.77736194488186	1.2440709237347	22.2581643657551	9.92454265835566
hsa-miR-548e-3p	0	1.2440709237347	6.54651893110444	1.04468870087954
hsa-miR-548i/miR-548c-5p/miR-548b-5p/miR-548d-5p/miR-548o-5p/miR-548as-5p/miR-548am-5p	15.8138445380577	13.6847801610817	8.51047461043578	0
hsa-miR-548k	4.51824129658791	2.48814184746941	0	3.13406610263863
hsa-miR-548o-3p	2.25912064829395	6.22035461867352	10.4744302897671	2.61172175219886
hsa-miR-548t-3p/miR-548aa	13.5547238897637	17.4169929322859	17.675601113982	25.5948731715488
hsa-miR-548v	6.77736194488186	6.22035461867352	8.51047461043578	6.26813220527726
hsa-miR-548z/miR-548h-3p	4.51824129658791	4.97628369493882	2.61860757244178	2.61172175219886
hsa-miR-550a-3-5p	47.441533614173	44.7865532544493	30.7686389761909	34.9970714794647
hsa-miR-550a-3p	135.547238897637	204.027631492491	121.110600225432	242.367778604054
hsa-miR-550a-5p	47.441533614173	42.2984114069799	29.45933518997	30.2959723255067
hsa-miR-551a	6.77736194488186	2.48814184746941	3.27325946555222	7.3128209061568
hsa-miR-551b-3p	72.2918607454065	197.807276873818	70.0477525628175	91.9326056773998
hsa-miR-551b-5p	0	4.97628369493882	0	0.522344350439772
hsa-miR-5584-5p	0	0	5.891867037994	3.13406610263863
hsa-miR-5585-3p	2.25912064829395	13.6847801610817	3.27325946555222	2.08937740175909
hsa-miR-561-3p	6.77736194488186	2.48814184746941	3.92791135866267	1.56703305131931
hsa-miR-561-5p	2.25912064829395	1.2440709237347	4.58256325177311	9.92454265835566

hsa-miR-5690	22.5912064829395	22.3932766272247	14.4023416484298	28.2065949237477
hsa-miR-5695	4.51824129658791	2.48814184746941	2.61860757244178	3.6564104530784
hsa-miR-5699-3p	0	7.46442554240822	11.1290821828776	5.74578785483749
hsa-miR-5699-5p	128.769876952755	46.030624178184	26.8407276175282	25.072528821109
hsa-miR-570-3p	15.8138445380577	11.1966383136123	34.6965503348536	8.35750960703635
hsa-miR-574-3p	33.8868097244093	34.8339858645717	120.455948332322	109.692313592352
hsa-miR-576-3p	24.8503271312335	12.440709237347	8.51047461043578	4.70109915395794
hsa-miR-576-5p	13.5547238897637	8.70849646614293	13.0930378622089	7.3128209061568
hsa-miR-577	13.5547238897637	12.440709237347	9.16512650354622	12.0139200601147
hsa-miR-579-3p	9.03648259317581	11.1966383136123	26.1860757244178	27.1619062228681
hsa-miR-579-5p	13.5547238897637	2.48814184746941	9.16512650354622	2.08937740175909
hsa-miR-582-3p	13.5547238897637	4.97628369493882	9.81977839665667	15.1479861627534
hsa-miR-582-5p	257.539753905511	68.4239008054087	316.196864372345	65.2930438049714
hsa-miR-584-3p	2.25912064829395	7.46442554240822	2.61860757244178	1.04468870087954
hsa-miR-584-5p	11.2956032414698	34.8339858645717	6.54651893110444	24.5501844706693
hsa-miR-585-3p	6.77736194488186	3.73221277120411	3.27325946555222	0
hsa-miR-585-5p	9.03648259317581	3.73221277120411	13.0930378622089	9.40219830791589
hsa-miR-589-3p	38.4050510209972	58.4713334155311	63.5012336317131	96.6337048313577
hsa-miR-589-5p	6.77736194488186	16.1729220085512	16.3662973277611	20.3714296671511
hsa-miR-590-3p	684.513556433068	775.056185486721	619.955342775591	234.532613347457
hsa-miR-590-5p	6436.23472698947	27110.7935700267	3059.18829650511	27071.540650242
hsa-miR-597-5p	65.5144988005246	106.990099441185	13.7476897553193	24.0278401202295

hsa-miR-598-3p	210.098220291338	404.323050213779	436.652812704666	1634.41547252605
hsa-miR-615-3p	11.2956032414698	32.3458440171023	9.81977839665667	23.5054957697897
hsa-miR-616-5p	0	2.48814184746941	5.891867037994	4.17875480351817
hsa-miR-619-5p	2.25912064829395	11.1966383136123	22.9128162588656	15.6703305131931
hsa-miR-624-5p	11.2956032414698	18.6610638560206	12.4383859690984	28.7289392741874
hsa-miR-625-3p	4.51824129658791	21.14920570349	30.1139870830804	66.8600768562908
hsa-miR-625-5p	33.8868097244093	49.7628369493882	33.3872465486327	75.7399308137669
hsa-miR-627-3p	6.77736194488186	3.73221277120411	14.4023416484298	2.08937740175909
hsa-miR-627-5p	13.5547238897637	22.3932766272247	25.5314238313073	76.7846195146464
hsa-miR-628-3p	36.1459303727032	36.0780567883064	32.7325946555222	71.0388316598089
hsa-miR-628-5p	27.1094477795274	17.4169929322859	13.7476897553193	25.5948731715488
hsa-miR-629-5p	225.912064829395	125.651163297205	116.528036973659	47.5333358900192
hsa-miR-641	24.8503271312335	57.2272624917964	13.7476897553193	34.4747271290249
hsa-miR-642b-3p/miR-642a-3p	15.8138445380577	1.2440709237347	6.54651893110444	3.13406610263863
hsa-miR-643	4.51824129658791	9.95256738987763	9.16512650354622	17.2373635645125
hsa-miR-651-3p	6.77736194488186	4.97628369493882	2.61860757244178	1.56703305131931
hsa-miR-651-5p	22.5912064829395	33.589914940837	5.891867037994	3.13406610263863
hsa-miR-652-3p	546.707196887137	243.837901052002	278.881706465049	113.87106839587
hsa-miR-652-5p	291.42656362992	218.956482577308	365.950408248738	253.337009963289
hsa-miR-654-3p	11.2956032414698	0	12.4383859690984	5.74578785483749
hsa-miR-660-3p	13.5547238897637	2.48814184746941	11.1290821828776	2.61172175219886

hsa-miR-660-5p	103.919549821522	146.800369000695	165.626928956942	285.722359690555
hsa-miR-664a-3p	9.03648259317581	3.73221277120411	12.4383859690984	9.40219830791589
hsa-miR-664a-5p	2.25912064829395	1.2440709237347	7.20117082421489	3.13406610263863
hsa-miR-671-3p	65.5144988005246	26.1254893984288	104.744302897671	45.9663028386999
hsa-miR-671-5p	1036.93637756692	855.920795529476	907.347523851076	1362.79641029736
hsa-miR-6720-3p	2.25912064829395	1.2440709237347	5.23721514488355	4.70109915395794
hsa-miR-6723-5p	0	0	5.891867037994	10.4468870087954
hsa-miR-675-3p	13.5547238897637	318.482156476084	6.54651893110444	248.635910809331
hsa-miR-675-5p	54.2188955590549	2312.72784722281	6.54651893110444	695.762674785776
hsa-miR-676-3p	11.2956032414698	12.440709237347	17.0209492208716	13.0586087609943
hsa-miR-6830-3p	4.51824129658791	0	0	0
hsa-miR-6852-5p	0	0	0	5.74578785483749
hsa-miR-6886-5p	0	1.2440709237347	0.654651893110444	7.3128209061568
hsa-miR-708-5p	126.510756304461	136.847801610817	41.243069265958	39.6981706334226
hsa-miR-7-1-3p	253.021512608923	319.726227399819	826.825340998491	834.183927652315
hsa-miR-744-3p	92.6239465800521	130.627446992144	89.0326574630204	124.840299755105
hsa-miR-744-5p	286.908322333332	747.686625164557	244.839808023306	507.196364277018
hsa-miR-758-3p	11.2956032414698	0	3.27325946555222	0
hsa-miR-7-5p	0	6.22035461867352	3.27325946555222	9.92454265835566
hsa-miR-760	0	12.440709237347	5.891867037994	7.3128209061568
hsa-miR-766-3p	13.5547238897637	49.7628369493882	18.9849049002029	39.6981706334226
hsa-miR-769-3p	2.25912064829395	3.73221277120411	13.0930378622089	37.6087932316636

hsa-miR-769-5p	153.620204083989	160.485149161777	80.5221828525847	90.8879169765202
hsa-miR-7706	6.77736194488186	4.97628369493882	4.58256325177311	4.17875480351817
hsa-miR-7974	51.9597749107609	33.589914940837	200.978131184906	89.8432282756407
hsa-miR-873-5p	9.03648259317581	11.1966383136123	17.675601113982	8.35750960703635
hsa-miR-874-3p	352.422821133857	523.75385889231	181.338574391593	521.299661738892
hsa-miR-874-5p	2.25912064829395	21.14920570349	15.7116454346507	19.8490853167113
hsa-miR-877-3p	0	7.46442554240822	0.654651893110444	3.6564104530784
hsa-miR-877-5p	29.3685684278214	77.1323972715517	26.8407276175282	39.6981706334226
hsa-miR-887-3p	6.77736194488186	0	5.891867037994	1.04468870087954
hsa-miR-92a-1-5p	63.2553781522307	80.8646100427558	60.8826260592713	63.7260107536521
hsa-miR-92a-3p	54.2188955590549	79.6205391190211	58.91867037994	120.661544951587
hsa-miR-92b-3p	133.288118249343	354.560213264391	37.3151579072953	146.778762473576
hsa-miR-92b-5p	29.3685684278214	34.8339858645717	17.0209492208716	27.1619062228681
hsa-miR-93-3p	67.7736194488186	106.990099441185	94.269872607904	225.652759389981
hsa-miR-934	117.474273711286	941.761689267171	43.8616768383998	547.93922361132
hsa-miR-935	1147.63328933333	1212.96915064134	1530.57612609222	1449.50557247037
hsa-miR-93-5p	4197.44616453016	5253.71151093166	10702.9038004627	23611.531672929
hsa-miR-939-3p	2.25912064829395	6.22035461867352	3.92791135866267	4.17875480351817
hsa-miR-939-5p	2.25912064829395	2.48814184746941	13.0930378622089	9.92454265835566
hsa-miR-9-3p	20.3320858346456	17.4169929322859	32.0779427624118	23.5054957697897
hsa-miR-940	24.8503271312335	313.505872781145	64.1558855248235	164.016126038088
hsa-miR-941	212.357340939632	175.414000246593	78.5582271732533	31.3406610263863

hsa-miR-942-3p	4.51824129658791	1.2440709237347	4.58256325177311	12.5362644105545
hsa-miR-942-5p	2.25912064829395	9.95256738987763	5.23721514488355	3.6564104530784
hsa-miR-95-3p	307.240408167978	490.163943951473	201.632783078017	686.36047647786
hsa-miR-9-5p	94.883067228346	104.501957593715	54.3361071281669	49.1003689413385
hsa-miR-96-3p	15.8138445380577	29.8577021696329	14.4023416484298	10.9692313592352
hsa-miR-96-5p	386.309630858266	1955.67949211095	638.940247675794	1002.37880849392
hsa-miR-98-3p	2.25912064829395	14.9288510848164	2.61860757244178	15.6703305131931
hsa-miR-98-5p	1554.27500602624	3163.67235905735	2205.52222788909	3353.45072982333
hsa-miR-99a-5p/miR-100-5p	6305.20572938842	2358.758471401	13908.0794691314	5211.95192868804
hsa-miR-99b-3p	646.108505412071	381.929773586554	1253.6583753065	418.397824702257
hsa-miR-99b-5p	3128.88209788712	2442.11122329122	1231.40021094075	785.083558710977
