# Supplementary material for: A comparison between SOLiD 5500XLand Ion Torrent PGM-derived miRNA expression profiles in two breast cell lines
Source: Genet Mol Biol. 2020 Apr 27;43(2):e20180351. doi: 10.1590/1678-4685-GMB-2018-0351 (PMC7201575; doi:10.1590/1678-4685-GMB-2018-0351)
Supplement: Table S4 - [file 1415-4757-GMB-43-2-e20180351-suppl6.pdf]

Supplementary Material to "A comparison between SOLiD 5500XL- and Ion Torrent PGM-derived miRNA expression profiles in two breast cell lines"

Table S4 - Nucleotide content and homopolymer counts of the more abundant miRNA (tables 2 and 3) found in PGM and Solid platform.

**PGM platform**								
miRNA	GC content	G content	C content	U content	A content	2-nucleotide repeat	3-nucleotide repeat	4-nucleotide repeat
3613-5p	0.23	0.14	0.09	0.73	0.05	3	1	1
4455	0.41	0.41	0.00	0.53	0.06	2	2	1
424-3p	0.39	0.22	0.17	0.26	0.35	3	1	0
16-1-3p	0.55	0.14	0.41	0.27	0.18	5	2	0
25-5p	0.41	0.14	0.27	0.32	0.27	3	3	1
20a-3p	0.41	0.23	0.18	0.32	0.27	3	0	0
let-7i-5p	0.48	0.30	0.17	0.22	0.30	2	1	
1296-5p	0.64	0.23	0.41	0.27	0.09	6	2	0
200c-3p	0.48	0.22	0.26	0.30	0.22	3	0	1
1307-5p	0.64	0.05	0.59	0.27	0.09	4	2	1
Total % of each base	0.46	0.20	0.26	0.34	0.19			
**Solid platform**								
150-5p	0.55	0.14	0.41	0.27	0.18	5	2	0
142-5p	0.33	0.14	0.19	0.19	0.48	2	2	0
223-3p	0.41	0.14	0.27	0.27	0.27	3	3	1
3607-5p	0.41	0.27	0.14	0.23	0.36	2	1	0
4284a	0.61	0.17	0.44	0.17	0.22	2	2	1
199a-3p/ 199b-3p	0.41	0.23	0.18	0.32	0.27	3	0	0
1249b	0.64	0.05	0.59	0.27	0.09	5	2	1

181b-3pb	0.38	0.14	0.24	0.19	0.43	4	0	0
29a-3p/29c-3p[a]	0.39	0.18	0.20	0.30	0.32	4.5	1.5	0
103a-3p	0.48	0.30	0.17	0.22	0.30	2	1	0
152-5p	0.48	0.22	0.26	0.30	0.22	3	0	0
4521	0.55	0.32	0.23	0.23	0.23	4	0	0
301b	0.39	0.22	0.17	0.26	0.35	3	1	0
107b	0.48	0.26	0.22	0.22	0.30	2	1	0
Total % of each base	0.44	0.19	0.25	0.25	0.28			

[a]Average of the sequences.
